# Water usage, hygiene and diarrhea in low-income urban communities—A mixed method prospective longitudinal study

**DOI:** 10.1016/j.mex.2019.11.018

**Published:** 2019-11-19

**Authors:** Rebeca Sultana, Charlotte Crim Tamason, Leela Sengupta Carstensen, Jannatul Ferdous, Zenat Zebin Hossain, Anowara Begum, Peter Kjær Mackie Jensen

**Affiliations:** a Copenhagen Center for Disaster Research, Section for Global Health, Department of Public Health, University of Copenhagen, Copenhagen, Denmark; b Institute of Health Economics, University of Dhaka, Dhaka, Bangladesh; c icddr,b, Dhaka, Bangladesh; d Department of Microbiology, University of Dhaka, Dhaka, Bangladesh

**Keywords:** Diarrhea, Water quantity, Domestic and personal hygiene, Qualitative research, Bangladesh

## Abstract

Epidemiological studies considered water use and hygiene practices as central risk factors for diarrhea. Few studies focused on independent association of water quantity with diarrheal diseases. This study aimed to describe the methodological protocol that adapted multidisciplinary and mixed-method research approach to assess how water usage including water quantity influences the attributable risk for diarrhea in a low-income urban community in Bangladesh.

The quantitative, anthropological and microbiological approaches were threaded together to provide a greater understanding of the infrastructural, behavioral and microbial interactions to fathom the dimensions of fecal oral transmission pathways within the households. The use of the *'Choleraphone'* (i.e. a mobile phone based real time diarrheal reporting system) was a contemporary approach intended to cut down on resources, reduce research fatigue and provide more accurate data compared to the 'gold standard' (i.e. visiting a household of diarrhea cases within 48 hours) for measuring diarrhea incidence. Development of methods to measure water quantity using qualitative and quantitative approach within a setting where meter water connection is rare was another unique feature of this protocol. This protocol provided guidance and insight on how multiple methods of different disciplines can be combined to enrich understanding of waterborne diseases.

## Graphical abstract

**Figure:**
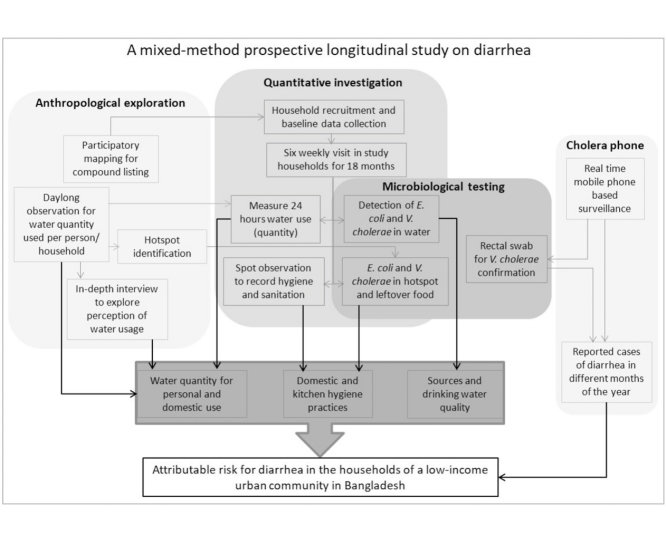

**Specification Table**

**Table:** 

Subject Area:	*Environmental Science*
More specific subject area:	*Assessment of diarrhea and water usage in low-income urban community*
Protocol name:	*Combating cholera caused by climate change*
Reagents/tools:	*The study used divers methods and tools following mixed method approach to conduct a prospective longitudinal study on water usage and diarrhea incidence*
*Experimental design:	*Prospective longitudinal study for 18 months in randomly selected 477 cohort households out of 13,621 households within a low-income urban setting*
Trial registration:	*Not applicable*
Ethics:	*The protocol has been internationally peer-reviewed before the submission to the Research Review Committee of icddr,b (International Centre for Diarrheal Disease Research, Bangladesh). The Ethical Review Committee (ERC) of icddr,b, Bangladesh reviewed and approved the study protocol.*

*Value of the Protocol

**Table:** 

• *The proposed methodological approach provided the opportunity for real time data collection on diarrheal incidence from community settings through a mobile phone based surveillance system which is a contemporary approach.* • *This study methodology helped to get a holistic understanding of diarrhea and its risk factors by incorporating multidisciplinary and mixed method research approach. In this study qualitative/anthropological research methods and findings were fed into quantitative, microbiological and epidemiological data collection to enrich the overall findings in a contextual manner.* • *This study provided a scientific way to measure the water quantity for personal and domestic hygiene within the community where an unmetered water connection is predominant.*

## Protocol data

• Given the current climate change projections, water stress (reduced water quality and quantity) is expected to increase in Bangladesh [1], which could lead to increased risk of waterborne diseases.
• Despite the global concern of waterborne infectious diseases, significant gap remained within the scientific community on epidemiology and transmission dynamics of these diseases. Although people's behaviour in terms of water use and hygiene practices is seen as a central risk factor of waterborne diarrheal disease [2,3], little attention is provided to know whether or not water quantity independently increases the risk of diarrheal diseases including describing the magnitude of this effect [4].
• Since the water and health outcomes are complex, interactive, nonlinear, and dynamic, and a multiplicity of different transmission pathways are avaiable to most of the waterborne pathogens, a comprehensive multidisciplinary mixed-method research approach is urged to address this major public health problem to provide high quality real time data in a holistic and contextual manner to enhance understanding of disease transmission.
• The methodology developed in this protocol will be useful to implement in future studies within different low-income countries to understand the disease dynamics that include complex behavioral, social and environmental exposure pathways of disease transmission.

## Description of protocol

### Overall objective of the study

To assess how water usage including availability and quantity for domestic and personal hygiene in different months of the year influence the attributable risk for diarrhea in households in a low-income urban community in Bangladesh.

### Study design

**Fig. 1:**
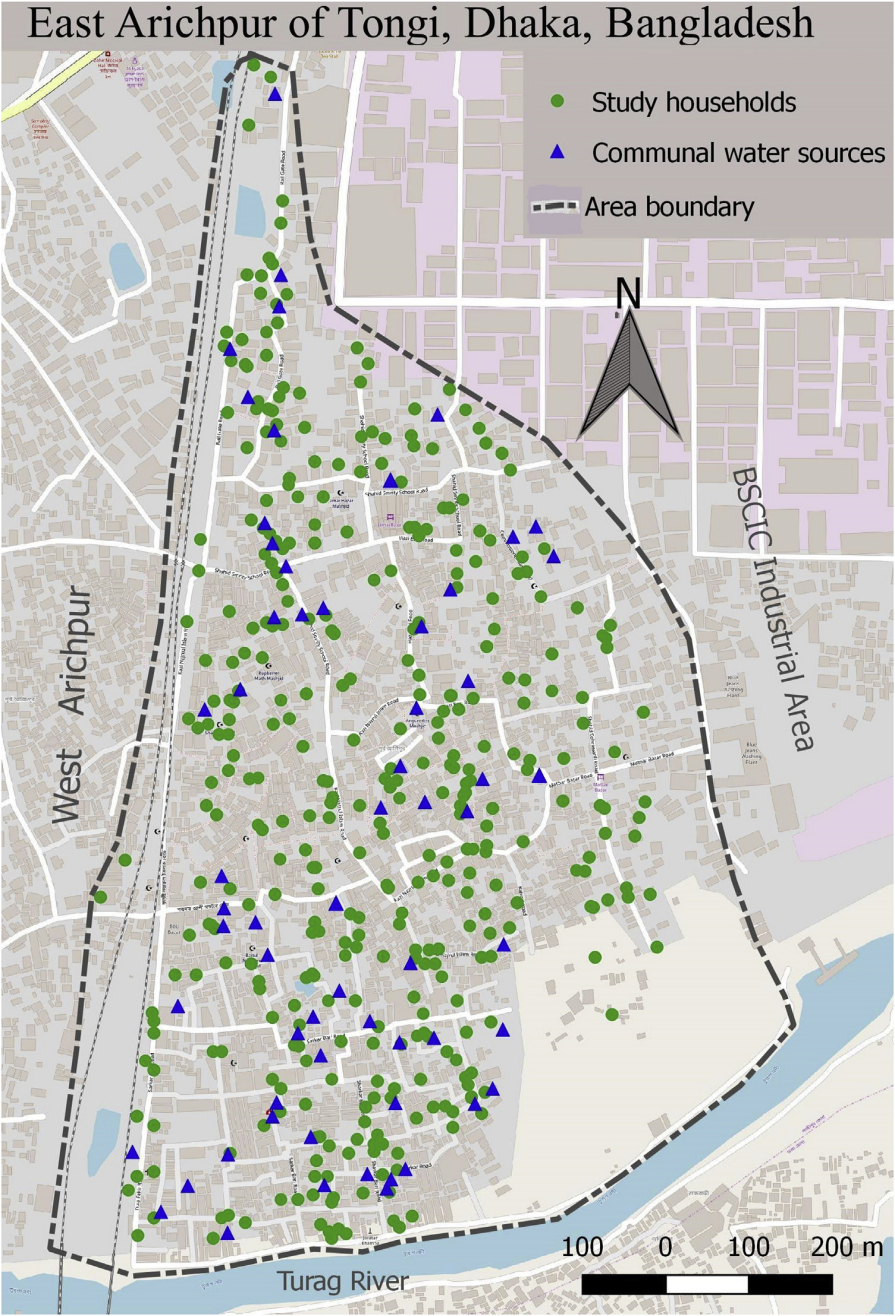
Study households and the communal water sources used by the study households of East Arichpur, Dhaka, Bangladesh from September 2014 to December 2015.

This proposed study was a prospective longitudinal study conducted in the East Arichpur urban area located northwest of Dhaka city in Bangladesh (Fig. 1) [5]. Most of the residents of this area were living within a low-income setting and was known to have high incidence of water-borne diseases including cholera [6,7]. This study adopted a mixed method approach to get in-depth understanding on water usage and diarrheal disease of the community people. Conducting a mixed-method study could be useful in such research where social and physical issues interact to create an understanding of the context and illustrate quantitative findings. This approach has been applied in several water-related studies [8,9]. Research related to water, hygiene and health is not only linked to human behaviour but also with the structural contexts in which the behaviour is embedded [10]. Incorporating a qualitative approach in this research could provide a greater in-depth insight to enhance the understanding [9,11].

### Selection of study households

The household was the unit of the study. This study used random sampling to recruit study households using the list of households of the community. A team trained in qualitative and quantitative data collection, conducted transect walk, informal group discussions and participatory mapping [12] to list all the households of East Arichpur community. The transact walk helped the team to identify number of *paras* (a small geographical cluster with name known to the residents) within the community, the size of each *para*, potential informants for group discussion and mapping, probable number of group discussion required in each *para* and suitable place and time of group discussion and mapping. The team conducted group discussions and mapping to understand the geographical layout of the area and identify clusters of household that share a common yard locally known as 'compound') using different sources of water for domestic use, living and sharing arrangement of water, sanitation and cooking facilities. The team also collected information on regularity of water availability and seasonal availability during this group discussion.

The participants of the group discussions were the residents who had knowledge about the area to draw the map of their small geographical segment where they lived. The total number of group discussion was decided based on the transect walk. In each group discussion five to ten participants were invited. One researcher facilitated the discussion and mapping exercise, one researcher took notes and another researcher observed and recorded the overall process. The research team and the participants drew the map collectively. Based on the mapping exercise, the team developed a list that included the information on cluster of households within a compound, water source, living and sharing arrangement of water, sanitation and cooking facilities (Table 1). The households living within a neighborhood or residential area with a clusters of households, predominantly sharing poor housing and shared water and/or sanitation and/or cooking facilities were used as inclusion criteria as these are used to characterize the low-income urban settings [13]. The team randomly chose the study households from the list using a random number generator from the Microsoft Excel file. The team enrolled a total of 477 households to cohort for 18 months from East Arichpur to achieve the study objective [5,14]. The required sample size for this study was 425 (details of sample size calculation in the 'STable-1').

**Table 1:** Characteristics compound^a^ and water sources of East Arichpur community, Bangladesh from June 2014 to December 2015.

Characteristics	Numbers
Number of Households	13,876
Number of compounds	1,437
Average number of person per household	4
Average number of household per compound	10
Missing information of compounds	117
**Type of sharing (number of compounds)**	(**n =** 1,320)
Share water point, kitchen and toilet	1,297
Share water point and toilet	14
Share kitchen and toilet	6
Missing information	3

**Compound structure**	
Semi *pacca* (wall concrete, roof tin or wood)	1,186
Concrete (wall and roof made with concrete)	115
Kachha (wall and roof tin or wood made)	10
Missing information	3

**Household structure**	
Nuclear family	1,295
Mess (single member of different families live within a household)	25

**Type of water sources in the compound**	
Private submersible pump	474
Municipal government submersible pump	828
Deep tubewell	14
Shallow tubewell	2
Concrete made borehole	2

**Location of water collection point**	
Inside of the compound	1,292
Outside of the compound	12
Both inside and outside of the compound	13
Missing information	3

a Compound is cluster of households sharing same yard and other facilities.

The team excluded the households that do not share resources (i.e., water or sanitation or cooking facilities) with other households as they did not fall into the low-income urban settings criteria. The team excluded the households if the head of household was unable to provide consent due to illness or disability or any household member refused to participate in the study. The team considered the household as dropped out if: the household migrated to outside of the study area or half of the household members' opt-out of the study or opt-out of diarrhea surveillance procedures. The dropped out household was replaced with another household randomly from the household list of the community.

### Baseline data collection

A research team member visited the selected households and asked for consent to participate in this study. Upon consent to participate, the research team set up an appointment to visit the family to complete baseline data collection at a specific time when all household members were expected to be at home (Table 2). After baseline, a mobile phone with a pre-paid SIM-card was given to each household to report occurrence of diarrhea within the household members. The last three-digits of the phone number served as the unique identification number for each household. Each household member was also assigned a unique identification number (phone number: - 2 digit and person code 01–20). To record the water quantity the researcher took measurement and photo of each water storage containers (Table 2). The research team obtained the coordinates of study households and communal water sources using a global positioning system (GPS). Q-GIS software was used to locate the sites on a Google map (Fig. 1).

**Table 2:** Issues explored through quantitative and qualitative data collection tools with the study households of East Arichpur, Bangladesh from June 2014 to December 2015.

Topic	Specific questions/issues explored
	**Baseline data collection**
	**Demographic information**
	Name of the *para*, How long have you been living here, how many rooms do you have, what materials are used to construct the wall and the roof, each of the household member's age, sex, education, occupation, relationship with household head and questions related to households wealth index and income per month

	**Water sources and usage**
**Water source**	Water source/s was/were used in last one year (tap/pipe/hand pump/river/well/pond/rainwater), if ground water, was it government or private (pump/well/ tubewell), presence of storage (roof tank/ground tank), flow of water for 24 hours continuous or interrupted, source shared with other household/s or not, is there any waiting time for water collection. Usage of water by each specific source (cooking, drinking, bathing, washing and cleaning).
**Storage of drinking water in household**	Is the drinking water treated, if yes how often, type of treatment (boiling, filter, chlorine, alum), water stored container used at household in last one month, Type of containers used (bucket/clay pot, plastic drum), size of the containers (in liter), handling of water (pour water, running from tap, dipped in container), cleaning information of container (how often, using which cleaning agent).
	**Cooking sites**
	How many households shared the same stove and cooking site
	**Sanitation and diarrhea last one year**

**Defecation practices**	Disposal of feces of young child <2 years, defecation practices of children 2–5 years, number of latrines household has access to, how many households share the latrine, probing of defecation when the latrine is occupied, is there any broken latrine.
**Diarrhea in last one year**	Is there any household member hospitalized in the last 1 year for diarrhea, Has anyone died from diarrhea, age of family member at death

	**Spot observation (household and compound premises)**
	Is there any feces visible in latrine or in the yard, Is there any stagnant water inside compound (common yard shared by multiple households), any handwashing facility in latrine, presence of soap or detergent in handwashing facility, is household floor clean (no presence of human/bird/animal feces or waste), is the compound floor clean.
**Kitchen utensils**	Plate, glass, mug and other utensils appear clean or not, is there any presence of fly in household cooking area
**Cleanliness of household members**	Hands of the present adult and children under five are clean or not (visible dirt, black fingernails), Does the person's cloth appear clean or not (visible fresh dirt, food, grime), is there any animal present in the compound (list of animals), do animals roam freely in household or compound

	**Photo and global positioning system (GPS)**
**Visual documentation**	Photo of room of the household, floor, latrine, water sources, each of the water storage containers of the household and cooking site, garbage and defecation points. GPS coordinates of all the study households.

	**Six weekly visit**
**Household member**	Change of any household member since last visit (age and sex of new member). Record if someone permanently left household since last visit.
**Water use**	Same source using since last visit, type of source and storage since last visit, is there more or less water available in the source since last visit, using more or less water daily compared to last visit. Change of container since last visit, if yes type and size of new container/s (in liter).
**Last 24 hours**	Flow of water in collection point within last 24 hours (24 hours available, less than 24 hours [include specific time/hour]), water collection point within last 24 hours, Was the drinking water clear or cloudy/colored/dirty, Was the water had good smell or bad smell, taste was better or not good, treatment of water last 24 hours.
**Food intake since yesterday**	List of foods have been eating by household member/s since yesterday (including fish, meet, rice, rice soaked in water, curry, lentil, raw vegetable, fruit, betel leaf and nut), time of eating (at breakfast, lunch, dinner), How many time food was cooked yesterday, If there is any food left today from yesterday to eat.

	**Diarrhea**
	Is there anyone in the household had diarrhea since last 2 days, if yes did they call to the *'Choleraphone'* center, if not why not.
**Photos**	Collect picture of the new container/s
	**Spot observation (household and compound premises)**
	(Same as baseline)

	**Water quantification of last 24 hours (using 'tally sheet')**
**Use of 'tally sheet'**	How many containers were used from last 24 hours, number of time filled the container/s from last 24 hours, was there any new container filled with water which is not recorded in '24 hours tally sheet' given to you, if yes record the type and size of container and number of times this new container/s was filled.
**Activities done without container**	What are the activities you/anyone of the household performed without using water from container in last 24 hours, how many times were the following activities done without using any container since last 24 hours (washing hands, bathing, cleaning dishes, washing cloth).

	**Household questionnaire for diarrhea affected person**
**Demography**	Age and sex of the patient, ID of the patient including household ID.
**Diarrhea**	How many times the person defecated loose stool in last 24 hours, how many days ago loose stool started, from what time of the day loose stool started, was the loose stool colorless or of any color, was it watery, did it contain blood/mucous, any symptoms of fever/vomiting/tiredness/fainting/cramping, did the patient visit a community clinic/hospital/pharmacy, was any diagnosis done, if yes what was the result, what type of medicine was taken during illness, did the patient stop going to work/school due to illness, if yes for how many days.
**Water use**	Did the patient use different water source since our last visit, if yes identify the place (identify source type), why did you change water source, what purpose you used the water source for.
**Food habit**	What type of food the patient ate before loose stool started (list from six-weekly questionnaire), did the patient eat any food where water was added.
**Rectal swab**	Was any rectal swab taken from patient
	**Qualitative exploration**
	**Day long observation**
**Water measurement**	Volume and frequency of water use for personal activity: hand, face, leg rinsing/washing, bathing, ablution, water use after urination and defecation Volume and frequency of water use for domestic activity: washing cooking utensils, cleaning kitchen, cleaning household premises, mopping house, cleaning toilet and bathroom. The team also recorded the changes in frequency, sources and quantity based on different months, and the reason behind such changes.

	**In-depth and follow-up interview**
**Individual water use practices**	Drinking, bathing, washing hand, face and legs, washing genital and anus after urination and defecation and reported quantity of water use in each of the household and personal activities.
**Changes of water use**	The changes in frequency, sources and quantity based on different season, and the reason behind such changes as well as practices of water use during extreme weather events; e.g., extreme heat, heavy and continuous rainfall, extreme cold or cold waves were recorded.
**Perception and reason for water stress**	What the informants perceived about water stress (i.e., water quantity and quality), reason for water stress, seasons of water stress, what were the coping strategies during water stress and what were the alternative sources of water during water stress (if any).
**Water usage for domestic purpose**	The team explored household activities that required water; cooking, washing vegetable, cleaning utensil, cleaning kitchen, cleaning leaving room and courtyard, cleaning toilet, and washing clothes only with the female members.

### Six-weekly follow-up visit

In every six-week interval, one researcher visited the cohort households and collected information on water usage, hygiene, sanitation practices and diarrhea episodes (Table 2).

#### Spot observation

In each of the visit the researcher conducted spot observation using a structured format and visual documentation to record actual practices hygiene and water use (Table 2). Any changes in water storage container also recorded during this visit.

#### 24 h water quantity measurement

The researcher also recorded the water consumption information by the household members within last 24 h using a 'tally sheet' that included a picture of all the water storage containers of the households (Figure S1). The research team provided a training of the household water collector to record water use in the first six-weekly visit. One day before the six-weekly visits, the researcher provided the 'tally sheet' to the household caretaker (i.e., the female or male family member who spent the most time in the house) to mark down with tally for each time each of the containers were refilled. The following day, the researcher again visited the household to collect the 'tally sheet'. During this visit, the researcher also conducted probing (i.e., seek elaboration of a person's response) following the 'tally sheet' to confirm the recorded water quantity. Any changes in the container or water sources were included in the 'tally sheet' in each visit. The team provided a training of the household water collector to record water use in the first six-weekly visit. The team recorded only frequency of activities that used running tap water. Later water quantity was measured using a proxy for quantity of each activities based on water quantity data that was collected as part of in-depth anthropological exploration in a subset of households (described in the in-depth anthropological exploration section below).

#### Water sample collection for microbiological testing

**Fig. 2:**
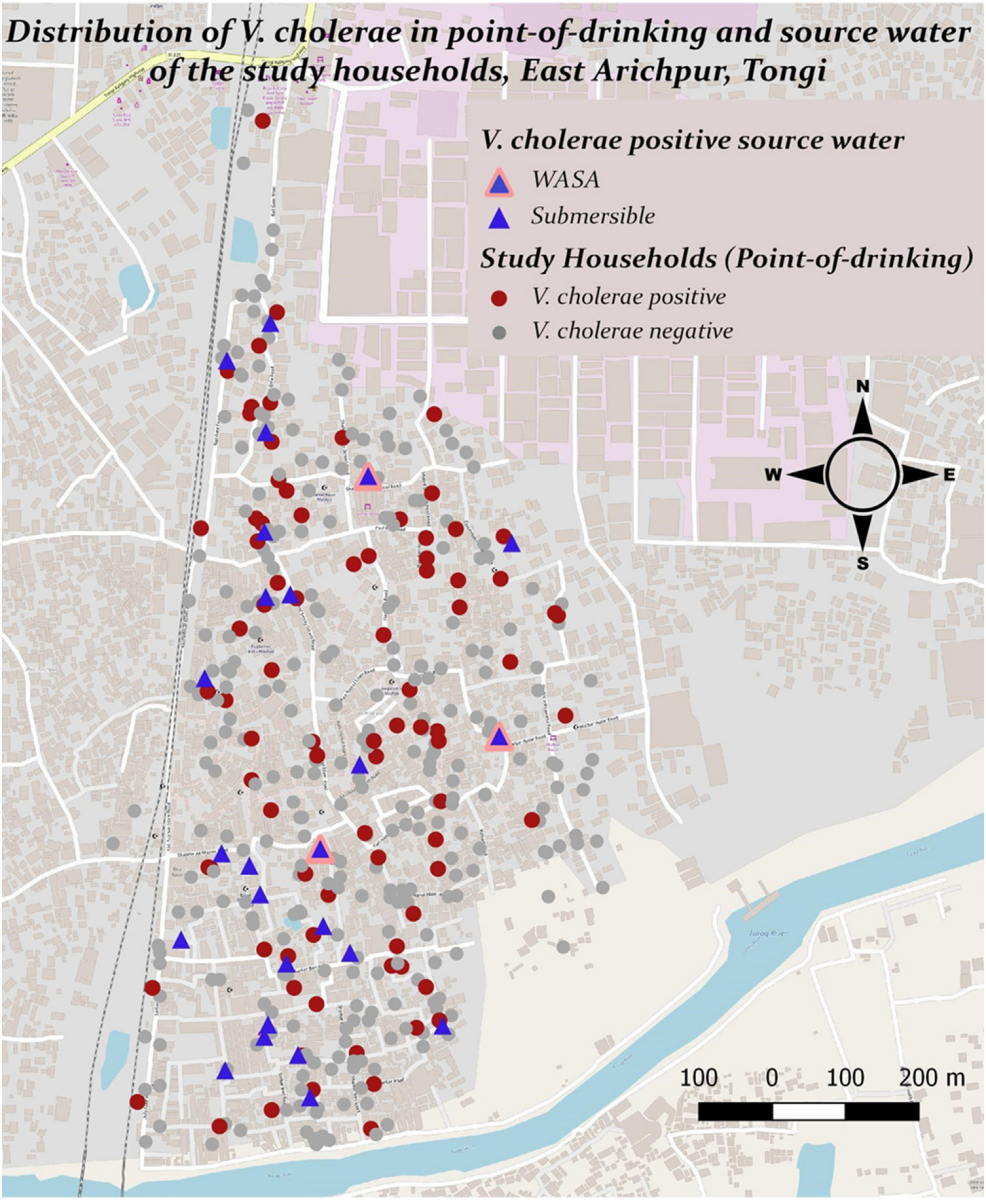
Distribution of *V. cholerae* positive households in household drinking water and communal sources of East Arichpur from September 2014 to October 2015.

To measure the water quality, the researcher also collected water sample from point-of-drinking vessels (i.e., a mug, glass, bottle or jug, that household members used to drink water) and communal sources tap stands used by each study household. The researcher collected a volume of 150−200 mL water sample in pre-sterilized wide-mouth water sampling bottles and transported in a cool box to the Environmental Microbiology Laboratory within two to four hours of collection. The microbiologist assessed the basic water quality by measuring colony forming units (CFUs) of fecal coliforms thermotolerant *Escherichia coli* (*E. coli*) by membrane filtration of 100 mL water samples according to the method described earlier [15]. More extensive virulence gene profile of *E. coli* was sought for characterization of pathogenic *E. coli* and their host source identification in a subsample of water sample (personal communication with Jannatul Ferdous). Presence of *Vibrio cholerae* (*V. cholerae*) in water samples was also examined in a subset of samples and the detail method and result is provided elsewhere [5]. Isolates of *V. cholerae* were also examined for detection of a range of virulence genes, antibiotic resistance genes and phylogenetic analysis. Twenty three percent (88/388) of households and 38 % (25/66) of source water were positive for *V. cholerae* at least once in the visits conducted at six-weekly interval (Fig. 2) [5].

* Source: Ferdous et al. [5].

### The real time ‘Choleraphone’ based surveillance to record diarrhea cases

#### Reporting of diarrhea

**Fig. 3:**
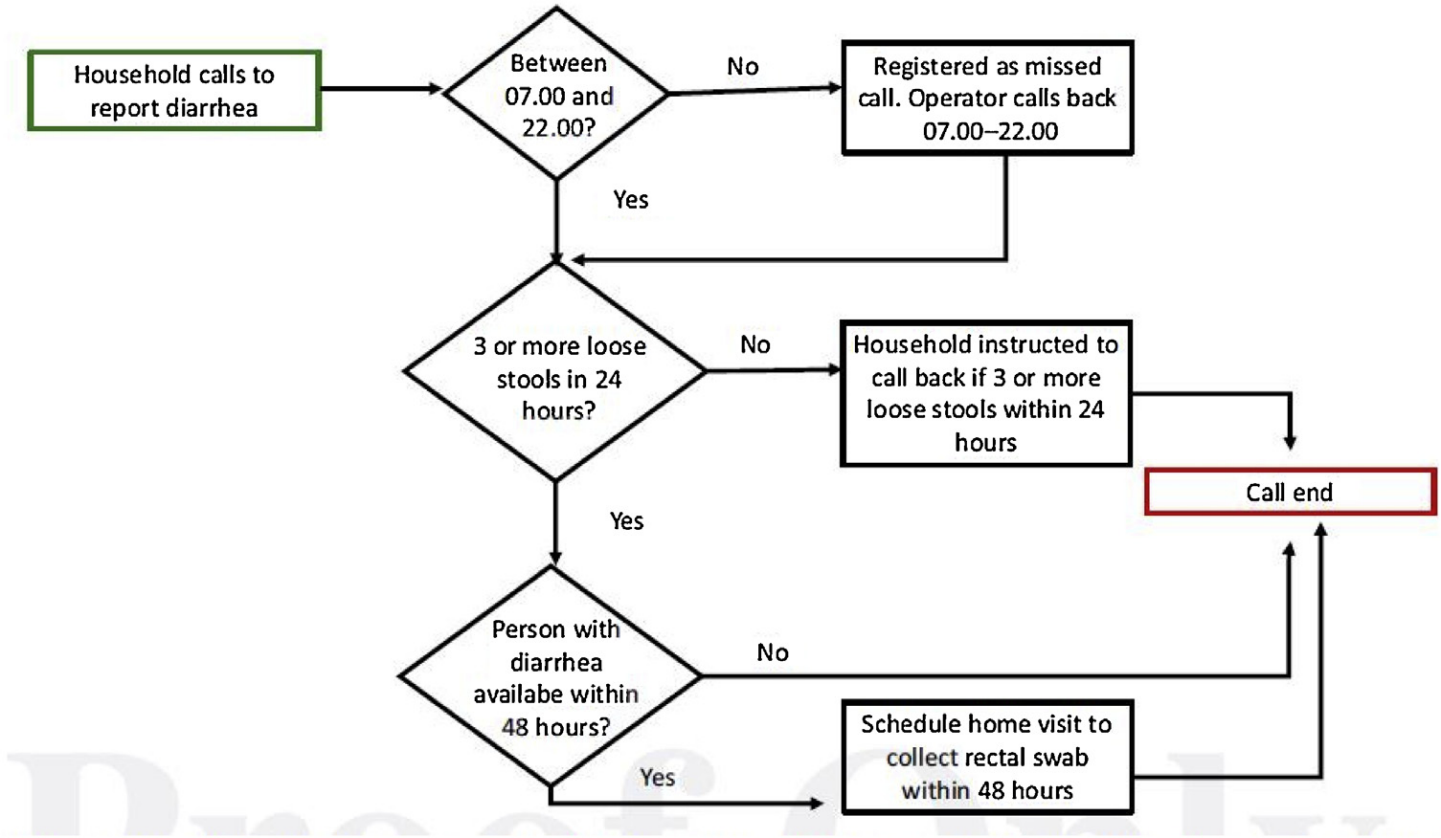
The real time diarrhea reporting system to record diarrhea cases from the community in the East Arichpur through the *'Choleraphone'* from June 2014 to December 2015.

The *'Choleraphone'* consisted of the mobile reporting system as the connection between the call center and the households where the diarrhea cases were identified (Fig. 3). Carstensen LS et al. (2019) published the detail methods of this phone-based diarrhea reporting system elsewhere [14]. A call center was established to answer the call of the study participants. The call center was open from 7:00 am to 10:00 pm seven days a week to receive any phone calls. If the household caretaker called after 10 o’clock at night, the staff returned the call in the following morning. Upon receiving a call, the call center staff first asked questions to identify if the person had three or more loose stools within 24 h which was defined as diarrhea according to World Health Organization [16]. Then staff asked the caller questions about stool type, duration and frequency, if treatment had been sought, and when the person would be available at home so a detailed questionnaire and rectal swab sample could be collected within 48 h.

* Source: Carstensen et al. [14].

During the baseline visit, the researcher provided a demonstration to the household caretaker including a laminated poster with instructions on how and when to call to the call center to report diarrhea. The study phone number was preprogrammed into the *'Choleraphone'* of the household [14]. The caretaker reported all diarrhea episodes for all household members using the *'Choleraphone'*. If the person with diarrhea was >11 years old, the caretaker would then pass the phone to the person with diarrhea to answer more sensitive questions about the diarrhea episode. If the person with diarrhea was not at home, the call center staff set up a time to call when the person would be available at home [14].

To remind the study household members to report diarrhea pre-recorded messages were sent out 1.5 weeks and 4.5 weeks and check-in phone call was made at 3.0 weeks between two 6-weekly visits During each round of the 6-weekly visits, each household was asked to show that they still had the mobile phone and that it was in functioning.

#### Household visit on diarrhea episodes and patient’s rectal swab sample collection

A researcher visited all households within 48 h that reported diarrhea through this surveillance to collect information on diarrhea episode (Table 2). Afterward, the researcher instructed the patient (or guardian if the patient was a child <11) how to take a rectal swab using a sterile swab, do two full turns in the rectum and place it directly into a sealable plastic tube containing Cary Blair medium (in grams per liter: sodium thioglycolate, 1.5 g; disodium phosphate, 1.1.g; calcium chloride, 0.1 g; sodium chloride, 5 g; agar, 5 g; pH 8.4) [17]. All the rectal swabs were then transported to the field lab and incubated in APW for 6 h and tested by dipstick for confirmation of *V. cholerae* O1 and O139 based on previous studies [[17], [18], [19], [20], [21]]. The test results were delivered to the patients' households through the *'Choleraphone'* within 24 h and they were given instructions for further medical treatment.

#### Evaluating the ‘*Choleraphone*’ using focus group discussion (FGD)

To improve the mobile phone surveillance system, a team of anthropologists conducted eight FGDs with the household members of selected cohort households to explore perception of diarrhea, barriers and advantages in using the *'Choleraphone'* to report diarrheal cases. Carstensen LS et al. (2019) published the detail methods of participants selection and the results elsewhere [14]. The team conducted separate FGDs with adult male and female group. To represent the geographical diversity, the team selected participants from different neighborhoods in East Arichpur.

**Fig. 4:**
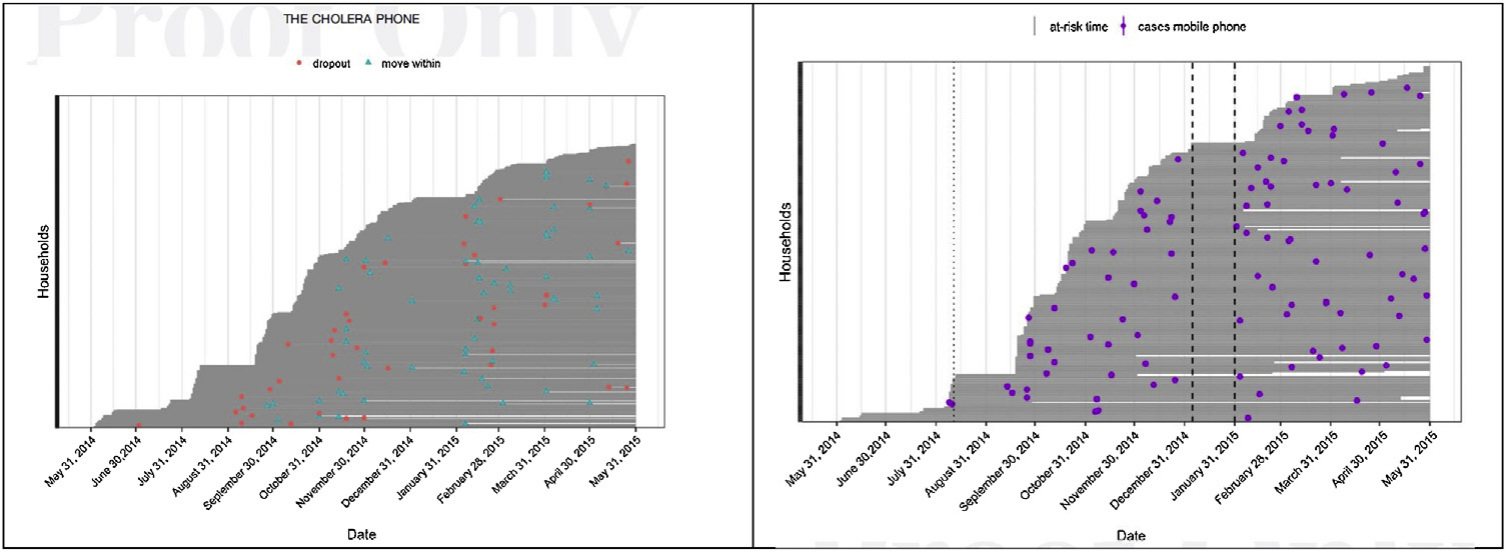
Cases of diarrhea reported through real time mobile phone based surveillance system in East Arichpur from May 2014 to May 2015.

After the focus group discussions, the *'Choleraphone'* surveillance system went through a number of changes. Based on the findings, the word *"diarrhea"* was replaced with "three or more loose stools in 24 h" as the term *'diarrhea'* was described in interchangeable terms with *'cholera'* [14]. Based on the findings, we also replaced the call center agent with the 'physician-led call-center' enabling the caller to receive general medical advice including diarrhea. During the household visits the team also gifted two packets of oral rehydration solution (ORS) to patients. During informal discussion at 6-weekly visits, the respondents reported feeling grateful that they could call a doctor for health-related questions free of charge from home. We provided 20 BDT (approximately US$ 0.25) for each call made by households for reporting diarrhea as reimbursement of call cost. Due to these changes the number of calls from the participants reporting diarrhea was increased (Fig. 4).

* Source: Carstensen et al. [14].

### In-depth anthropological exploration in a subset of cohort households

A team of trained anthropologists collected data using ethnographic approach. The researchers first built good rapport with the community and study households to gain trust. The researchers roamed in the entire community for the first two months and conducted several small groups and individual informal discussions to become acquainted with the community and gain an understanding of the existing water usage and management structure and practices. From the preliminary field exploration, the team identified household with 24 h water availability and households <24 h water availability. Thus, the team categorized households into two groups based on the availability. Within this each group, the team identified 'ease water use' method (i.e., using running water from tap) and 'difficult water use' method (i.e., using small water container for example mug to pour water from water storage and/or using hand pump). Thus, the team categorized households in four groups: '24 h available and ease method', '24 h available difficult method', '<24 h water available ease method' and '<24 h water available difficult method' (Table 3). Based on this different water availability and method, and willingness of participants of the study household, the team purposively selected the 24 households (six from each group) to cover differences in community residents’ perception and practices attributable to differences in water usage and availability.

**Table 3:** Data collection for qualitative exploration in a subset of households of East Arichpur, Bangladesh from September 2014 to June 2016.^a^

Data collection tools	24 hours water availability		Without 24 hours availability		Total
	Ease method	Difficult method	Ease method	Difficult method	
Different water user group	Ease and available	Available but not ease	Ease but not available	Not ease not available	
Enroll household	6	6	6	6	**24**
In-depth interview (female)	6	6	6	6	**24**
In-depth interview (male)	3	3	3	3	**12**
Observation (days)	18	18	18	18	**72**
Follow-up interview	18	18	18	18	**72**

a The timeline is different in this table from other tables as this indicates different data collection period of this particular qualitative component.

In these 24 households, the team conducted day-long observation, semi-structured follow-up interviews and in-depth interviews and to get in-depth insight about the water usages and hygiene practices. The team used audio tape recorder to collect data for all the interviews.

#### Observation

To measure the quantity of water usage for each type of domestic and personal purposes and to collect information about changes in practices across and within different seasons, the team conducted bimonthly (once every other month) observations over the course of one year (Table 3). The 12 households were selected based on the availability and willingness of the participants to allow us to perform a daylong observation to record water usage for 24 h. In each household, six daylong observations were conducted in one year. Two team members stayed in the study household for the whole day (from early morning to night) and performed this observation. During observation, the team measured the quantity of water use by each member for each type of personal activities and domestic activities (Table 2). The team used a measured bucket and/or a measured mug before/after the activities to measure the volume of water of each activities of the household members. The team used a stopwatch to measure volume for the activities using running water.

To attenuate the observation bias [22,23] the team spent substantial time in the study households and in the community to desensitizing their presence as outsiders. To attenuate the observer bias [22], the first author provided intensive training to all the observers so that they can have the same understanding.

#### Follow-up interview

After the daylong observation, the team conducted follow-up interviews in the observed households to record the reported water use of two more days within the same week of observation. This follow-up interview was used to cross check the findings of observation and to record changes in frequency, sources and quantity of water for each activity for three days.

#### In-depth interview

To obtain an in-depth understanding of the perception and practices on individual and domestic water use, water stress and coping strategies (Table 2), the team conducted in-depth interview with one adult female member of each of 24 households and one adult male member from 12 of these 24 households (Table 3). The team included more female participants than male, since women are the main custodians of domestic water and responsible for its usage. The team interviewed the leading female member of the household who was responsible for water management activities. The male participants were selected based on their availability at home and willingness. The duration of each interview was 120−150 min. The team collected data in two to three sessions when the participant is not willing to provide information in one sitting. When the team found child member/s (up to 12 years) in the household, they explored with the mothers about child's personal water use practices.

Besides the in-depth interviews and observation, the team also spent time within the community throughout the data collection period to obtain information of any communal event of activities linked to water usage and infrastructure. As part of this activity the team conducted several FGDs, key information interviews (KII), and informal individual and group discussions with community residents to obtain historical information and changes in water usage practices within the community.

#### Hotspot identification

During daylong observation, the team also identified potential hotspots within household including kitchen and sanitation facilities. Hotspots are places of possible transmission (as contaminations spots on the fecal oral transmission route, i.e., from the latrine to the mouth) where one or more household members can transmit bacteria from one person to the other or “receive” bacteria from the external environment: from food and kitchen implements. The household hotspots are surfaces that are suspected to be contaminated with fecal material (latrine door, water pump handle, kitchen knife, glasses, plates) and/or blood or body parts of fish (any type of fish particularly shellfish and sea fish) chicken, and any kind of meat due to frequent touching, infrequent cleaning, and/or poor hygiene. The team identified places that had hand contact with surfaces immediately after coming out of the toilet. The team also observed the food processing and cooking activities to identify possible contaminated kitchen implements. The team also listed the ‘leftover’ food item that was preserved for more than six hours.

#### Sample collection for microbiological testing of hotspots and leftover food

During six-weekly visit, environmental swab samples or “hotspot” samples among 32 households (average 16 households each month) were collected from 4 hotspots of direct exposure within household domain: cutting knife, food plate, latrine doorknob, drinking water vessel surface. Hotspot surface samples were obtained by swabbing approximately 10 cm^2^ area with sterile cotton swab stick soaked with Phosphate Buffer Saline (PBS) and the swab sticks were returned to the tube containing 3 ml of PBS. All samples of leftover/pre-cooked rice, eggs, lentils, meats, fish and shellfish that had been resting at room temperature or warmer for more than 6 h were taken as because the conditions were found conducive for reaching infective levels (more than 10^6^ cells) of *V. cholerae* [24].To collect solid food sample, 25 g food was collected in sterile bag. In case of liquid food, 25 mL of liquid was taken. All the samples were kept at 4 °C in a sterile cool box and transferred to the Environmental Microbiology Laboratory of University of Dhaka within 4 h of collection for microbiological testing. All the samples were examined for fecal thermotolerant *E. coli* contamination by quantifying CFU in chromogenic selective media (mTEC) for thermotolerant *E. coli*. Detection of *V. cholerae* prevalence was conducted by targeting the species-specific gene *ompW* in PCR.

### Data analysis

#### Quantitative data

We used descriptive statistics mean, range, standard deviation to measure the water quantity liter per capita per day (LCPD) for each household. To categorize the households with different water usage group, the four categories of water usage group was considered which was defined as part of in-depth anthropological exploration (Table 3). The annual diarrhea incidence rate (IR) was calculated for the cases reported for diarrhea during data collection period. The incidence rate ratio (IRR) was estimated including the 95 % confidence interval. We planned to assess the possible association between quantity of water usage and diarrhea incidence using regression analysis. We also planned to perform univariate risk analysis on diarrhea incidence and the known risk factor of diarrhea.

#### Qualitative data

The interviewers transcribed the audio-recorded data. The team expanded the observation field notes and typed in ‘Microsoft word’ for soft copy. After note expansion and transcription, each researcher prepared a code list separately considering the objectives and emerging theme of the study. The team members sat together with their code lists and prepared a final code list. All data were coded manually using the code list. Coded data was summarized according to the study objectives and relevant themes. The team thoroughly reviewed all summarized data to understand the relationships between different themes and subthemes. Later on comparison and triangulation were made among the different tools and findings. Triangulation is important for the rigor of qualitative research [25]. This analysis produced data on interconnectedness among seasonality, water stress and hygiene behavior/water usage in terms of quantity.

#### Microbiological data

A descriptive analysis was performed for households where we portrayed the percentage of households that had *E. coli* and *V. cholerae* contamination in drinking water, leftover food and hotspot surfaces. The proportions of water samples positive for *E. coli* and *V. cholerae* in point-of-drinking and communal source water were calculated. Logistic regression test was employed to examine the association of *E. coli* and *V. cholerae* between point-of-drinking and sources, treated and non-treated drinking water, drinking vessel type. A subset of hotspot samples was further analyzed by PCR to detect the virulence factors of five different diarrheagenic pathotypes of *E. coli.* From PCR positive samples, *V. cholerae* strains were isolated for further genotypic and phenotypic characterization. For both *E. coli* and *V. cholerae*, seasonal prevalence of contamination was estimated in hotspot and food samples.

## Ethics

Informed written consent was obtained from all the study households. The data collection team obtained written informed consent from all the participants or their guardians (for children) included in this study. The protocol has been internationally peer reviewed before the submission to the Research Review Committee (RRC) of icddr,b. The Ethical Review Committee (ERC) of icddr,b, Bangladesh reviewed and approved the study protocol.

## Additional information

There were some changes made during field work due to field situation and field experience as outlined below. This information would be useful for future replication of the protocol.

• In the initial design, participatory mapping was planned to be implemented. During the data collection period the team identified that using this method for entire mapping was not possible due to limited time and availability of the community residents. In an urban setting, individuals are less available compared to a rural setting. Thus the team employed both participatory mapping and mapping by the research team members by visiting door-to-door to complete the household listing.
• Identifying incidence of cholera was one of the specific objectives of this study but we found only eight cases of *V. cholerae* through rectal swab sample out of 281 reported diarrhea cases. We found some reasons for low incidence which are as follows: a) our qualitative exploration identified that community people have common practices of taking medication (e.g. anti-diarrheal or antibiotic) without consulting with a formal health practitioner when they realize that they having loose stools. b) Despite of thorough mock training on rectal swab sample collection, the team made incorrect use of 70 % ethanol (70 % ethanol is anti-microbial) for moistening the rectal swabs, and set the wrong temperature for the incubation for first few samples and we excluded these samples from our analysis. Later on, we provided hands-on training to the team for rectal swab sample collection.
• For anthropological exploration, in-depth interview was planned to be the first method of exploration. However, in first few days of rapport building, the team identified that the study households were biased by the exposure of quantitative baseline data collection and tended to inform the researcher that 'they did not have any diarrhea', 'they washed their hands always' and 'keep themselves clean always'. Thus, the team decided to conduct the observation as a first method so that they could build a good relationship with the study households and then conducted the in-depth interview as the second method.

The detail baseline questionnaire (STable 2), six-weekly visit questionnaire (STable 3), diarrhea reporting questionnaire (STable 4) and in-depth interview guideline (STable 5) were included as supplementary documents.

## CRediT authorship contribution statement

**Rebeca Sultana:** Conceptualization, Methodology, Investigation, Writing - original draft, Data curation, Visualization, Project administration, Funding acquisition. **Charlotte Crim Tamason:** Conceptualization, Methodology, Data curation, Formal analysis, Writing - review & editing, Project administration, Funding acquisition. **Leela Sengupta Carstensen:** Conceptualization, Methodology, Investigation, Data curation, Formal analysis, Writing - review & editing, Visualization, Project administration. **Jannatul Ferdous:** Conceptualization, Methodology, Investigation, Data curation, Formal analysis, Writing - review & editing, Visualization, Project administration. **Zenat Zebin Hossain:** Conceptualization, Methodology, Investigation, Data curation, Formal analysis, Writing - review & editing, Visualization, Project administration. **Anowara Begum:** Resources, Writing - review & editing, Supervision. **Peter Kjær Mackie Jensen:** Conceptualization, Methodology, Resources, Writing - review & editing, Supervision, Funding acquisition.

## Declaration of Competing Interest

Authors have no conflict of interest
