# Supplementary material for: Water usage, hygiene and diarrhea in low-income urban communities—A mixed method prospective longitudinal study
Source: MethodsX. 2019 Nov 19;6:2822–37. doi: 10.1016/j.mex.2019.11.018 (PMC6909126; doi:10.1016/j.mex.2019.11.018)
Supplement: Supplementary file 1 [file mmc1.docx]

| Household ID (or holding number) | __ __ __ | | |
| --- | --- | --- | --- |
| Date | Start :_ _ __-__ __-__ __ | | End: ___-__ __ |
| Time | Start :_ __ | | End __ |
|  |  | | |
| **Please see the following pictures of your water collection vessels** | **Capacity of the vessel in liter** | **How many times the vessel was filled (Add tally for each time)** | |
| 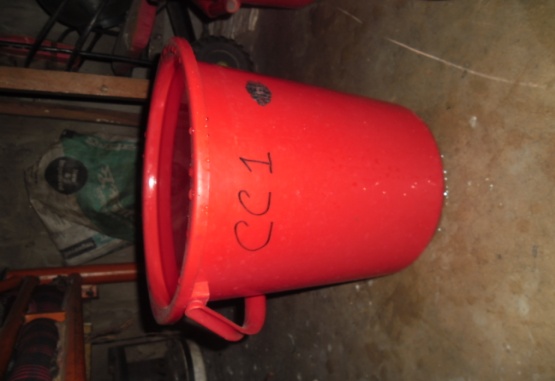CC1 | 14 liters |  | |
| C2  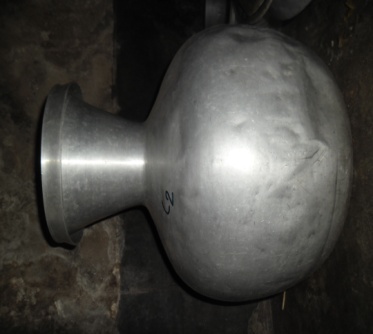 | 17 liters |  | |
| 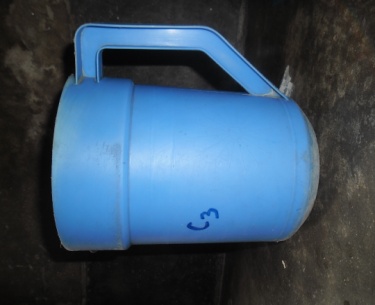C 3 | 3 liters |  | |
| CC3  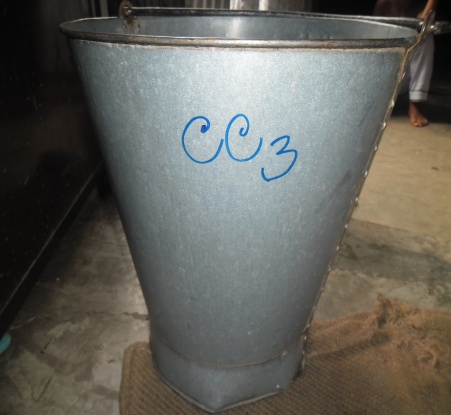 | 23 liters |  | |

The work done without any vessel: for example using tubewell or tap for washing hands

| **Please see the work in the picture** | **How many times the work was done (Add tally for each time)** |
| --- | --- |
| 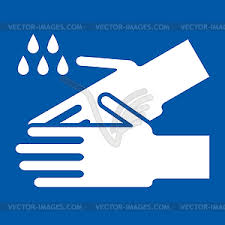Wash hands |  |
| 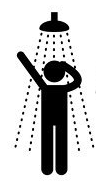Adult bath (Age 5+) |  |
| 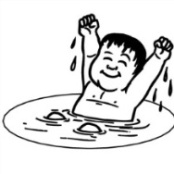Child bath (under 5 years) |  |
| 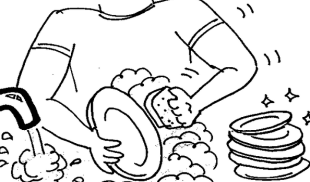Wash dishes |  |
| 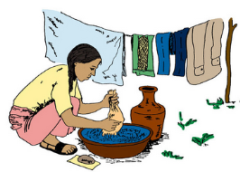Wash clothes |  |

The activities of household member did outside the home. Please ask this question to the member who were outside home and performed any of the following activities

| **Please see the work in the picture** | **How many times the work was done (Add tally for each time)** |
| --- | --- |
| 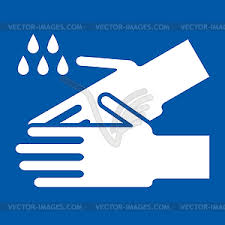Wash hands |  |
| 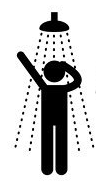Adult bath (Age 5+) |  |
| 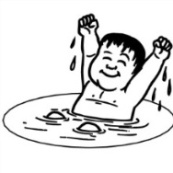Child bath (under 5 years) |  |
| 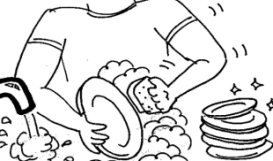Wash dishes |  |
| 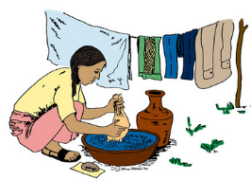Wash clothes |  |
