# Supplementary material for: Water usage, hygiene and diarrhea in low-income urban communities—A mixed method prospective longitudinal study
Source: MethodsX. 2019 Nov 19;6:2822–37. doi: 10.1016/j.mex.2019.11.018 (PMC6909126; doi:10.1016/j.mex.2019.11.018)
Supplement: Supplementary file 2 [file mmc2.docx]

| **Water usage, hygiene and diarrhea in low-income urban communities - a mixed method prospective longitudinal study** |
| --- |
| **Sample Size Calculation**  To ensure power to the primary outcome of the study, which is to estimate the association between diarrheal incidence and quantity of water use, the number of people needed enrolled in the study (n) is calculated from diarrheal longitudinal prevalence.  Arichpur has approx. 130,000 people. Based on the exclusion criteria (see section 5.3.3), we expect that 65,000 people (50%) will be left eligible for the study. We use a model designed by Schmidt et al. [1] to calculate sample size using 80%, a 95% confidence interval. assuming that incidence of diarrhea in the high water use group is 3.24, relative risk between the high and low water use group is 2.0 [2], and longitudinal prevalence in the control arm is .042, the sample size required for our study would be 60 people.  To account for clustering, we will use n=4.5 for intra-household clustering and n=60 for intra-compound clustering. We will inflate by 0.1 for intra-household and by 0.02 for intra-compound clustering. Therefore, n = 212.5 people for each tail or 425 total |
| ***Estimating the association between cholera incidence and water quantity***  Using a 400 household sample, we expect to have 1900 people as our sample size (4.5 per household), so we can calculate the power to estimate the association between cholera incidence and water quantity. Because cholera is less common than general diarrhea and is also acute, sample size is calculated using incidence.  Estimated inflation factor for cholera inter-household = .12 for clustering in households and .04 for clustering in compounds [3]. Therefore Deff = 1.42 + 3.36 = 3.78  Using conservative estimates based on unpublished research from Arichpur (ES Gurley, personal communication), and assuming that 80% of cholera cases are moderate to mild [4, 5], we expect to see 692 cholera cases per every 100,000 individuals and the resulting power would be 0.32. This gives us a 32% chance of finding significant results for cholera. |

**References**

1. Schmidt WP, Genser B, Barreto ML, Clasen T, Luby SP, Cairncross S, et al. Sampling strategies to measure the prevalence of common recurrent infections in longitudinal studies. Emerg Themes Epidemiol. 2010;7(1):5. doi: 10.1186/1742-7622-7-5. PubMed PMID: 20678239; PubMed Central PMCID: PMCPMC2922204.

2. van der Hoek W, Feenstra SG, Konradsen F. Availability of irrigation water for domestic use in Pakistan: its impact on prevalence of diarrhoea and nutritional status of children. Journal of Health, Population and Nutrition. 2002:77-84.

3. Teddlie C, Yu F. Mixed methods sampling: A typology with examples. Journal of mixed methods research. 2007;1(1):77-100.

4. WHO. Cholera Geneva: World Health Organization; 2014 [cited 2014 11 July]. Available from: <http://www.who.int/mediacentre/factsheets/fs107/en/index.html>.

5. Weil AA, Khan AI, Chowdhury F, Larocque RC, Faruque AS, Ryan ET, et al. Clinical outcomes in household contacts of patients with cholera in Bangladesh. Clinical infectious diseases : an official publication of the Infectious Diseases Society of America. 2009;49(10):1473-9. doi: 10.1086/644779. PubMed PMID: 19842974; PubMed Central PMCID: PMCPMC2783773.
