# Supplementary material for: Water usage, hygiene and diarrhea in low-income urban communities—A mixed method prospective longitudinal study
Source: MethodsX. 2019 Nov 19;6:2822–37. doi: 10.1016/j.mex.2019.11.018 (PMC6909126; doi:10.1016/j.mex.2019.11.018)
Supplement: Supplementary file 3 [file mmc3.docx]

International Center for Diarrhoeal Disease Research, Bangladesh (icddr,b)

**Baseline Visit of Water Use, Hygiene and Diarrhea Incidence in Arichpur, Tongi**

**FACE SHEET**

|  |
| --- |

|  |  |
| --- | --- |

**Cluster Number (1 to 20): Holding Number:**

|  |  |  |
| --- | --- | --- |

|  |
| --- |

**Block Number (A, B, C, D): Household Identification Number:**

|  |  |  |
| --- | --- | --- |

**Random Number**

**PLEASE COMPLETE ALL THE QUESTIONS USING BLUE OR BLACK INK ONLY**

**PLEASE STATE ALL GPS COORDINATES TO 4 DECIMAL PLACES**

**PLEASE MAKE SURE ALL THE ANSWER CODES/NUMBERS ARE WRITTEN IN ENGLISH**

**IF YOU FACE ANY DIFFICULTIES PLEASE CONTACT BIMAL K DAS (SFRO) ON *01712503646***

| **IDENTIFICATION** | | | |
| --- | --- | --- | --- |
| **Q No.** | **English Title** | **Answer** |  |
| **Q 1.0** | **GPS Coordinates (*to 4 decimal places*)** | **North** |  |
|  |  | **East** |  |
| **Q 2.0** | **Start Time of Data Collection**  [Hour(s):Minute(s)] | ☐☐ :☐☐ |  |

| **SECTION 1: DEMOGRAPHIC INFORMATION** | | | | |
| --- | --- | --- | --- | --- |
| **Q M1** | **I am going to start by asking you some general questions about you and your household.** |  | | |
| **Q 3.0** | **What is your name?** |  | | |
| **Q 4.0** | **What para do you live in?**  **DO NOT WRITE ARICHPUR/EAST ARICHPUR** |  | | |
| **Q 5.0** | **How long have you lived here?**  *In months* | ☐☐ **Months** | | |
| **Q 6.0** | **How many rooms are in your current house?** | ☐☐ **Rooms** | | |
| **Q 7.0** | **What materials are used to construct the walls?**  *Multiple options possible* |  | | |
|  | **Answer in English** | **Y/N = 1/0** | | **Skip To** |
| **Q 7.1** | Corrugated Metal |  | | **N/A** |
| **Q 7.2** | Concrete |  | |  |
| **Q 7.3** | Wood |  | |  |
| **Q 7.4** | Bamboo |  | |  |
| **Q 7.5** | Plastic |  | |  |
| **Q 7.6** | Others |  | | ***If NO, skip to Q 8.1*** |
| **Q 7.6 Other** | Other Name | **________________** | | **N/A** |
| **Q 8.0** | **What materialis the roof made of?**  *Multiple options possible* |  | | |
|  | **Answer in English** | **Y/N = 1/0** | | **Skip To** |
| **Q 8.1** | Corrugated Metal |  | | **N/A** |
| **Q 8.2** | Concrete |  | |  |
| **Q 8.3** | Wood |  | |  |
| **Q 8.4** | Bamboo |  | |  |
| **Q 8.5** | Plastic |  | |  |
| **Q 8.6** | Others |  | | ***If NO, skip to Q 9.0*** |
| **Q 8.6 Other** | Other Name | **________________** | | **N/A** |
| **Q 9.0** | **Does your household (or any member of your household) have?** | | | |
|  | **Answer in English** | **Y/N/UK=1/0/999** | | **Skip To** |
| **Q 9.1** | Electricity |  | | **N/A** |
|  | | | **[Number]** |  |
| **Q 9.2** | Almirah or Wardrobe [Number] | ☐☐ | | **N/A** |
| **Q 9.3** | Tables [Number] | ☐☐ | |  |
| **Q 9.4** | Chair or Bench[Number] | ☐☐ | |  |
| **Q 9.5** | Watch or Clock [Number] | ☐☐ | |  |
| **Q 9.6** | Khat (Cot) [Number] | ☐☐ | |  |
| **Q 9.7** | Chouki [Number] | ☐☐ | |  |
| **Q 9.8** | A radio that is working, not a phone radio [Number] | ☐☐ | |  |
| **Q 9.9** | Black and white television that is working [Number] | ☐☐ | |  |
| **Q 9.10** | Colour television that is working [Number] | ☐☐ | |  |
| **Q 9.11** | Refrigerator [Number] | ☐☐ | |  |
| **Q 9.12** | Bicycle (*used for commercial purposes not toy for children*)[Number] | ☐☐ | |  |
| **Q 9.13** | Motorcycle [Number] | ☐☐ | |  |
| **Q 9.14** | Sewing Machine [Number] | ☐☐ | |  |
| **Q 9.15** | Land Phone [Number] | ☐☐ | |  |
| **Q 9.16** | Showcase [Number] | ☐☐ | |  |
| **Q 9.17** | DVD Player [Number] | ☐☐ | |  |
| **Q 9.18** | Mobile Phones [Number] | ☐☐ | |  |
| **Q 9.19** | Computer/Laptop [Number] | ☐☐ | |  |
| **Q 9.20** | Other __________________________ | ☐☐ | |  |

| **Q 9.21** | **Which brands of mobile phones?[Number]** | **[Number]** | **Skip To** |
| --- | --- | --- | --- |
| **Q 9.21.1** | Maximus [Number] | ☐☐ | **N/A** |
| **Q 9.21.2** | Nokia [Number] | ☐☐ |  |
| **Q 9.21.3** | Samsung [Number] | ☐☐ |  |
| **Q 9.21.4** | Symphony [Number] | ☐☐ |  |
| **Q 9.21.5** | HTC [Number] | ☐☐ |  |
| **Q 9.21.6** | LG [Number] | ☐☐ |  |
| **Q 9.21.7** | Apple (iPhone) [Number] | ☐☐ |  |
| **Q 9.21.8** | Other Name __________________________ | ☐☐ | ***If NO, skip to Q10.0*** |
| **Q 9.21.9** | Other Name__________________________ | ☐☐ |  |
| **Q 9.21.10** | Other Name __________________________ | ☐☐ |  |
| **Q 10.0** | **By whom is the house occupied?** | **Answer = [ ]** | |
|  | **Answer in English** | **Answer Code** | **Skip To** |
|  | Nuclear family | **1** | ***Q 11.0*** |
|  | Multiple families (*extended family*) | **2** |  |
|  | Unrelated persons | **3** |  |
|  | Nuclear family with one or more related person | **4** |  |
|  | Others | **777** | **N/A** |
| **Q 10.0 Other** | Other (*Specify*) | _____________________________________ | |

| **Q 11.0** | ***Questionnaire for each individual in the household.***  ***Please fill this out for every household member*** | | | | | | |
| --- | --- | --- | --- | --- | --- | --- | --- |
| **Q 11.1**  **ID No.**  **Adult over 18 years = 01,02,03…**  *= 01, 02, 03...*  **Child 17 and under = 11, 12, 13…**  *=11, 12, 13...* | **Q 11.2**  **Name:** | **Q 11.3**  **What is this person’s sex?**  **1= Male**  **2= Female**  **3= Transgender** | **Q 11.4**  **How old is he/she?**  **(Years)**  **999= Unknown**  **666= Decline** | **Q 11.5**  **What is his/her relationship to the household head?**  **1= Self**  **2= Spouse**  **3= Parent**  **4= Child**  **5= Other Family Member**  **6= Non-family Member**  **999= Unknown**  **666= Decline** | **Q 11.6**  **What is his/her occupation?**  **See attached occupation code plan** | **Q 11.7**  **Does she/he know how to read?**  **1=Yes** (  **0=No** | **Q 11.8**  **How many years of formal education has he/she had?**  **0= None**  **_______ Years** |
|  |  |  |  |  |  |  |  |
|  |  |  |  |  |  |  |  |
|  |  |  |  |  |  |  |  |
|  |  |  |  |  |  |  |  |
|  |  |  |  |  |  |  |  |
|  |  |  |  |  |  |  |  |
|  |  |  |  |  |  |  |  |
|  |  |  |  |  |  |  |  |
|  |  |  |  |  |  |  |  |
|  |  |  |  |  |  |  |  |
|  |  |  |  |  |  |  |  |
|  |  |  |  |  |  |  |  |
|  |  |  |  |  |  |  |  |
|  |  |  |  |  |  |  |  |

| **Q 12.0** | **What is your average household income per month**? **This is the total income of all household members from their jobs.**  **(Bangladeshi Taka, BDT)***Any money sent or received to or from family members that do not live in the household is considered remittances.* | ☐ | ☐ | ☐ | | ☐ | ☐ | ☐ |
| --- | --- | --- | --- | --- | --- | --- | --- | --- |
| **Q 12) A** | **How much of that money do you send/receive in remittances per month?** | | | | | | | |
| **Q 12) A.1** | BDT Sent (e.g., to family in Bangladesh) | ☐ | ☐ | ☐ | | ☐ | ☐ | ☐ |
| **Q 12) A.2** | BDT Received | ☐ | ☐ | ☐ | | ☐ | ☐ | ☐ |
| **Q 12) A.3** | *If remittances received less than monthly, amount per year* | ☐ | ☐ | ☐ | | ☐ | ☐ | ☐ |
| **Q 12) A.4** | *If remittances sent less than monthly, amount per year* | ☐ | ☐ | ☐ | | ☐ | ☐ | ☐ |
| **Q 12) B** | **Do you receive any other things like rice, pulse, etc except money?** | **Answer = [ ]** | | | | | | |
|  | **Answer in English** | **Answer Code** | | | **Skip To** | | | |
|  | Yes | **1** | | | ***If YES, complete Q 12) B SPECIFY*** | | | |
|  | No | **0** | | | ***If NO, complete Q 12) C*** | | | |
| **Q12) B Specify** | *Specify* | **______________________________________** | | | | | | |
| **Q 12) C** | **Are you paying any loans right now?** | **Answer = [ ]** | | | | | | |
|  | **Answer in English** | **Answer Code** | | | **Skip To** | | | |
|  | Yes | **1** | | | **N/A** | | | |
|  | No | **0** | | |  |  |  |  |
| **Q 12) D** | **How much are you paying per month in loans?**  **For NO loans please put '000000'.** | ☐ | ☐ | ☐ | | ☐ | ☐ | ☐ |

| **SECTION 2: WATER SOURCES** | | | | | |
| --- | --- | --- | --- | --- | --- |
| **Q M2** | **Now I am going to ask you a series of questions about the sources of water you use for different purposes in your household.** Considering different purposes that you have collected water for, think about the purpose for which the largest quantity was used. Where did you collect this largest quantity of water from?  *(After going through the source where most water is taken)*  Do you use water from any other sources for any purposes during other times of the year? ***Probing****: What about when the seasons change or if the water is disconnected? Then what do you do? Do you ever use river, pond or rainwater?If yes, fill out a new row in the table for each additional source.* | | | |  |
| **Q13. Source ID**  **1 = Source from which most water is collected over last year**  **2** = **2^nd^ Source**    **3** = **3^rd^ Source**    **4** = **4^th^ Source** | **Q14. What is the source?**  **1 = Tap**  **2 = Pipe**  **3 = Hand Pump Vertical Pressure (Tubewell)**  **4 = Hand Pump Horizontal Pressure (Tubewell)**  **5 = Well (with bucket)**  **6 = River** (Skip to question 19)  **7 = Rainwater** (Skip to question 19)  **8 = Pond** (Skip to question 19)  **777 = Other, please specify…….** | **Q 14) A.Is it WASA or a type of groundwater?**  **1 = WASA Supply**  **2 = Individual Submersible Pump**  **3 = Communal Submersible Pump**  **4 = Well** (<100 ft)  **5 = Shallow Tubewell (100-250ft)**  **6 = Deep Tubewell (>250ft)**  **7 = Compressor Pump (>100,<250ft)**  **777 = Others**  **888 = N/A**  **999 = Unknown** | **Q 15) Is there a tank for storage?**  **0 = No Tank**  **1 = Roof Tank**  **2 = Ground tank with attached tap**  **4 = Ground tank without attached tap**  **3 = In-ground tank that pumps into roof tank**  **5 = In-ground tank with bucket**  **6 = In-ground tank with tubewell**  **777 = Other, please specify…….**  **888 = N/A** | **Q16. Is there water flowing and ready for collection 24-hours per day from this source?**  **1 = Yes, 7 days per week**    **0 = No, shut offs every day**  **2 = Usually available 24 hours, shut offs occasionally** | |
|  | **Other** |  |  |  | |
|  | **Other** |  |  |  | |
|  | **Other** |  |  |  | |
|  | **Other** |  |  |  | |
| **Q 13 Notes: (*If recent change has occurred*)** | |  | | | |

| **Source ID (continuation from Q13: 1,2,3,4...)** | **Q17. 0**  **Do you share the water source you use with other households?**  **1 =Yes**  **0= No**  ***If no, skip to Q19.0*** | **Q17.1**  **If yes, how many households are sharing the source in their own compound?**  **999=Unknown** | | **Q17.2**  **If yes, how many households are sharing the source in their Neighbouring compound?**  **999=Unknown** | | **Q18.0**  **Do you ever have to wait in line to use it (in minutes)?**  **1= Yes, how long**  **0**= **No**  **999=Unknown** | | |
| --- | --- | --- | --- | --- | --- | --- | --- | --- |
|  |  |  | |  | |  | **Time** | |
|  |  |  | |  | |  | **Time** | |
|  |  |  | |  | |  | **Time** | |
|  |  |  | |  | |  | **Time** | |
| **Q 19.0 What is the water used for?** | | **Source ID 1**  **1= Yes**  **0= No** | **Source ID 2**  **1= Yes**  **0= No** | | **Source ID 3**  **1= Yes**  **0= No** | | | **Source ID 4**  **1= Yes**  **0= No** |
| **19.1 Drinking** | |  |  | |  | | |  |
| **19.2 Cleaning the house** | |  |  | |  | | |  |
| **19.3 Bathing children** | |  |  | |  | | |  |
| **19.4** **Bathing self** | |  |  | |  | | |  |
| **19.5** **Washing hands** | |  |  | |  | | |  |
| **19.6 Washing plates/utensils** | |  |  | |  | | |  |
| **19.7 Washing clothes** | |  |  | |  | | |  |
| **19.8 Cooking** | |  |  | |  | | |  |
| **19.9** **Sanitation** | |  |  | |  | | |  |
| **19.10** **Adding water to leftover food** | |  |  | |  | | |  |
| **19.11 Preparing tea** | |  |  | |  | | |  |
| **19.12 Gargling** | |  |  | |  | | |  |
| **19.13 Ablution** | |  |  | |  | | |  |
| **19.14 Other** | |  |  | |  | | |  |

| **Q 20.0** | **Is the drinking water treated?**  This could be filtering, boiling, alum or other methods.It does not include using cloth, sari or meslun as a filter. | **Answer = [ ]** | |
| --- | --- | --- | --- |
|  | **Answer in English** | **Answer Code** | **Skip To** |
|  | Yes | **1** | **N/A** |
|  | No | **0** | ***Q 23.0*** |
| **Q 21.0** | **How often?** | **Answer = [ ]** | |
|  | **Answer in English** | **Answer Code** | **Skip To** |
|  | 1- 3 times in a week | **1** | **N/A** |
|  | 4-6 times in week | **2** |  |
|  | 7 or more times in a week | **3** |  |
|  | Unknown | **999** |  |
|  | Decline | **666** |  |
| **Q 22.0** | **How is the drinking water treated?** *Multiple answers possible* |  | |
|  | **Answer in English** | **Y/N = 1/0** | **Skip To** |
| **Q 22.1** | Boiling |  | **N/A** |
| **Q 22.2** | Alum |  |  |
| **Q 22.3** | Chlorine |  |  |
| **Q 22.4** | Filter |  |  |
| **Q 22.5** | Boil and Filter |  |  |
| **Q 22.6** | Other |  | ***If NO other, skip to Q23.0*** |
| **Q 22.6 Other** | Other name | _______________ | **N/A** |
| **Q 23.0** | **During the last month have you stored water in a container?** | **Answer = [ ]** | |
|  | **Answer in English** | **Answer Code** | **Skip To** |
|  | Yes | **1** | **N/A** |
|  | No | **0** | ***Q 31.0*** |

| **Q 24.0**  **Container ID** | | **Q 25.0**  **What is the type of the containers?**  **1= Plastic Bucket**  **2= Aluminum/Silver Bucket**  **3= Aluminum/Silver Kolshi**  **4= Clay Kolshi**  **5= Plastic Drum**  **6= Pitcher**  **7= Plastic Water Bottle**  **8 = Glass Water Bottle**  **777= Other, specify** | | **Q 26.0**  **Size in Liters (L):**  **500 ml = 0.5L**  **1000 ml = 1 L** | **Q 27.0**  **Handling of water**  1= **Poured out**  **2= Reused**  **3= Object dipped in container to remove water (e.g. a cup)** | **Q 28.0**  **Do you clean the containers?**  **1=Yes**  **0= No**  ***If NO, skip to***  ***Q 31.0*** | **Q 29.0**  **How often are the containers cleaned?**  **1= Daily**  **2=Weekly**  **3= Less than 1 time per week**  **0= Never** | **Q 30.0**  **How are the containers cleaned? (can choose more than** 1)  **1= Soap**  **2= Hand**  **3= Scrub Brush**  **4= Towel**  **5= Sari**  **6= Ash**  **7= Sand with detergent powder**  **8= Sand**  **9= Detergent/Vim**  **10 = Coconut Scrub**  **777= Other** | |
| --- | --- | --- | --- | --- | --- | --- | --- | --- | --- |
| **C1** |  |  | **Other _________** | **_________** L |  |  |  |  | **Other _________** |
| **C2** |  |  | **Other _________** | **_________** L |  |  |  |  | **Other _________** |
| **C3** |  |  | **Other _________** | **_________** L |  |  |  |  | **Other _________** |
| **C4** |  |  | **Other _________** | **_________** L |  |  |  |  | **Other _________** |
| **C5** |  |  | **Other _________** | **_________** L |  |  |  |  | **Other _________** |
| **C6** |  |  | **Other _________** | **_________** L |  |  |  |  | **Other _________** |
| **C7** |  |  | **Other _________** | **_________** L |  |  |  |  | **Other _________** |
| **C8** |  |  | **Other _________** | **_________** L |  |  |  |  | **Other _________** |
| **C9** |  |  | **Other _________** | **_________** L |  |  |  |  | **Other _________** |
| **C10** |  |  | **Other _________** | **_________** L |  |  |  |  | **Other _________** |
| **C11** |  |  | **Other _________** | **_________** L |  |  |  |  | **Other _________** |
| **C12** |  |  | **Other _________** | **_________** L |  |  |  |  | **Other _________** |
| **C13** |  |  | **Other _________** | **_________** L |  |  |  |  | **Other _________** |
| **C14** |  |  | **Other _________** | **_________** L |  |  |  |  | **Other _________** |
| **C15** |  |  | **Other _________** | **_________** L |  |  |  |  | **Other _________** |
| **C16** |  |  | **Other _________** | **_________** L |  |  |  |  | **Other _________** |
| **C17** |  |  | **Other _________** | **_________** L |  |  |  |  | **Other _________** |
| **Note: Were there uses or recycling not captured by the questions?** | | | | |  | | | | |

| **SECTION 4: COOKING SITES** | | | |
| --- | --- | --- | --- |
| **Q 31.0** | **How many households share the stove?** | ☐☐ |  |

| **SECTION 5: SANITATION** | | | |
| --- | --- | --- | --- |
| **Q 32.0** | **How do you dispose feces of young children < 2 years?** *Multiple answers possible* |  | |
|  | **Answer in English** | **Y/N=1/0** | **Skip To** |
| **Q 32.1** | In latrine |  | **N/A** |
| **Q 32.2** | In designated spot inside household |  |  |
| **Q 32.3** | In designated spot outside household |  |  |
| **Q 32.4** | Anywhere around the household |  |  |
| **Q 32.5** | Other |  | ***If NO, skip to Q32.6*** |
| **Q 32.5 Other** | Other (*Specify*) | **_________________________________________** | |
| **Q 32.6** | Not applicable (no children under 2 years) |  | **N/A** |
| **Q 32.7** | Decline |  |  |
| **Q 33.0** | **Where does your child between 2-5 years old defecate?**  *Multiple answers possible* |  | |
|  | **Answer in English** | **Y/N=1/0** | **Skip To** |
| **Q 33.1** | In latrine |  | **N/A** |
| **Q 33.2** | In designated spot inside household |  |  |
| **Q 33.3** | In designated spot outside household |  |  |
| **Q 33.4** | Anywhere around the household |  |  |
| **Q 33.5** | Other |  | ***If NO, skip to Q 33.6*** |
| **Q 33.5 Other** | Other specify | **_________________________________________** | |
| **Q 33.6** | Not applicable (no children between 2-5 years) |  | **N/A** |
| **Q 33.7** | Decline |  |  |

| **Q 34.0** | **How many latrines does your household have access to?** | ☐☐ | |
| --- | --- | --- | --- |
| **Q 35.0** | **Is/are the latrine shared, i.e., one household vs. multiple households?** | **Answer = [ ]** | |
|  | **Answer in English** | **Answer Code** | **Skip To** |
|  | Yes | **1** | **N/A** |
|  | No | **0** |  |
|  | Unknown | **999** |  |
| **Q 36.0** | **How many households share the latrine?** | ☐☐ | |
| **Q 37.0** | **Is any defecation occurring elsewhere if latrine is occupied?** | **Answer = [ ]** | |
|  | **Answer in English** | **Answer Code** | **Skip To** |
|  | Yes | **1** | **N/A** |
|  | No | **0** | ***Q 40.0*** |
|  | Unknown | **999** | **N/A** |
|  | Decline | **666** |  |
| **Q 38.0** | **If yes, where?**  *Multiple answer could come* | **Answer = [ ]** | |
|  | **Answer in English** | **Answer Code** | **Skip To** |
|  | In designated spot inside household | **1** | **N/A** |
|  | In designated spot outside household | **2** |  |
|  | Anywhere around the household | **3** |  |
|  | In a plastic bag | **4** |  |
|  | Other | **777** | ***Fill out Q38.0 OTHER*** |
| **Q 38.0 Other** | Other name | **_____________________________________** | |
|  | Decline | **666** | **N/A** |

| **Q 39.0** | **Who is defecating elsewhere?**  *Multiple answers possible* |  | |
| --- | --- | --- | --- |
|  | **Answer in English** | **Y/N=1/0** | **Skip To** |
| **Q 39.1** | Child in household |  | **N/A** |
| **Q 39.2** | Adult in household |  |  |
| **Q 39.3** | Neighbor’s child |  |  |
| **Q 39.4** | Adult neighbor |  |  |
|  | **Probing about defecation at night time or when the shared latrine is occupied.** | | |
| **Q 39.0** | **Notes:** | | |
| **Q 40.0** | **Is your latrine or any of the available latrines for your householdunimproved,e.g., crack in ringslab, broken seal, hanging latrine, crack or leaks in the base?** | **Answer = [ ]** | |
|  | **Answer in English** | **Answer Code** | **Skip To** |
|  | Yes, all of the latrines that are available are broken | **1** | **N/A** |
|  | Yes, some of them | **2** |  |
|  | No | **0** | ***Q42.0*** |
|  | Unknown | **999** | **N/A** |
| **Q 41.0** | **Are people using any of the broken latrines?** | **Answer = [ ]** | |
|  | **Answer in English** | **Answer Code** | **Skip To** |
|  | Yes | **1** | **N/A** |
|  | No | **0** |  |
|  | Unknown | **999** |  |

| **Q 42.0** | **Has anyone in your family been hospitalized for diarrhea in the last 1 year?** | **Answer = [ ]** | |
| --- | --- | --- | --- |
|  | **Answer in English** | **Answer Code** | **Skip To** |
|  | Yes | **1** | **N/A** |
|  | No | **0** | ***Q 44.0*** |
| **Q 43.1** | Personal Identification Numbers |  | |
| **Q 43.2** | Personal Identification Numbers |  | |
| **Q 43.3** | Personal Identification Numbers |  | |
| **Q 43.4** | Personal Identification Numbers |  | |
| **Q 43.5** | Personal Identification Numbers |  | |
| **Q 44.0** | **Has anyone in your family died from diarrhea in the last year?** | **Answer = [ ]** | |
|  | **Answer in English** | **Answer Code** | **Skip To** |
|  | Yes | **1** | **N/A** |
|  | No | **0** | ***Q45.0*** |
| **Q 44) A** | **Age of family member at death?** | **Years:**  ☐☐ | **Months:**  ☐☐ |

| **MEASUREMENTS FOR SECTION 3** | | | |
| --- | --- | --- | --- |
| **Q 45.0**  **Water source (this should match exactly with *Q 13.0*)**  **1= Where you collect most of your water**  **2= 2^nd^Source**  **3= 3^rd^Source**  **4= 4^th^ Source** | **Q 46.0**  **Can you show me the place where you collect most of your water** (  ***Distance from front door of household to source where most water is taken (if inside household, then 0)***  *Lv*  ***If less than 20 meters take measurement (in meters)***  **__________________________________ Meters**  ***[If more than 20 meters, GPS coordinates Q46) A****]* | **Q 46) A**  ***Distance from front door of household to source***  ***GPS coordinates (if more than 20 meters away):***  ***North***: *_________________* ***East***: *_________________* | |
|  | **____________________________________ *Meters*** | *_________________* ***North*** | *__________________* ***East*** |
|  | **____________________________________ *Meters*** | *_________________* ***North*** | *__________________* ***East*** |
|  | **____________________________________ *Meters*** | *_________________* ***North*** | *__________________* ***East*** |
|  | **____________________________________ *Meters*** | *_________________* ***North*** | *__________________* ***East*** |
|  | **____________________________________ *Meters*** | *________________* ***North*** | *__________________* ***East*** |

| **WATER METER INSTALLMENT ON TAPS & PIPES** | | | | |
| --- | --- | --- | --- | --- |
| **Q 47.0** | ***How many water meters need to be installed? Ask the family to show you all of the taps and pipes that they use as a water point (if any, please alert Bimal at the end of the day, so he can make a list of these households)*** | ☐☐ | |  |
| **Q 48.0** | **What type of floor is in the household?** *Multiple answers possible* | **Y/N/O=1/0/777** | **Skip To** |  |
| **Q 48.1** | Dirt or clay |  | **N/A** |  |
| **Q 48.2** | Cement |  |  |  |
| **Q 48.3** | Other |  | ***Complete Q48.3; OTHER*** |  |
| **Q 48.3 Other** | Other name | **________________________________________** | |  |

| **MEASUREMENTS FOR SECTION 5** | | |
| --- | --- | --- |
| **Q 49.0** | The distance between the front door of the household and the latrines (*in meters)*  *[****If more than 20 meters, GPS coordinates Q49) A****]* | **__________________________________ Meters** |
| **Q 49) A** | ***Please only put the number to 4 decimal places.***  ***North:***  ***East:*** | ***North:*** |
|  |  | ***East:*** |
| **Q 50.0** | **BLANK SPACE** | |
| **Q 50) A** |  |  |

| **OBSERVATIONS FOR SECTION 5** | | | | | |
| --- | --- | --- | --- | --- | --- |
| **Q 51.0** | **Are human feces visible in latrine?** | **Answer = [ ]** | | | |
|  | **Answer in English** | **Answer Code** | **Skip To** | | |
|  | Yes | **1** | **N/A** | | |
|  | No | **0** |  |  |  |
| **Q 52.0** | **Are there any stagnant water or puddles inside the compound?** | **Answer = [ ]** | | | |
|  | **Answer in English** | **Answer Code** | **Skip To** | | |
|  | Yes | **1** | **N/A** | | |
|  | No | **0** |  |  |  |
| **Q 53.0** | **Is there a bucket or tap with water in it for handwashing in latrine?** | **Answer = [ ]** | | | |
|  | **Answer in English** | **Answer Code** | **Skip To** | | |
|  | Yes, there is water | **1** | ***Q 55.0*** | | |
|  | No, there is a bucket or tap but without water | **2** | **N/A** | | |
|  | No, there is no bucket or tap | **0** |  |  |  |
| **Q 54.0** | **Is bucket, tap or handpump (with water available for 24 hours) for handwashing within 5 meters of latrine?**  (Ask: Can you show me where you wash your hands after using the latrine?) | **Answer = [ ]** | | | |
|  | **Answer in English** | **Answer Code** | | **Skip To** | |
|  | Yes | **1** | | **N/A** | |
|  | No | **0** | |  |  |
| **Q 55.0** | **Is there soap, soapy water or detergent available for handwashing after using the latrine?**  *If soap is not visible,* can you bring me your soap or detergent that you normally use for washing your hands after using the latrine | **Answer = [ ]** | | | |
|  | **Answer in English** | **Answer Code** | | **Skip To** | |
|  | Yes | **1** | | **N/A** | |
|  | No, neveruse soap after latrine | **0** | |  |  |
|  | Other | **777** | | ***Complete Q55.0; OTHER*** | |
| **Q 55.0 Other** | Other name | **________________________________________** | | | |
| **Q 56.0** | **Is the household floor clean?**  *Clean means that there are no feces, human or animal,on the ground and that any garbage is in a pile over to the side in corner of the household or in a container.Garbage is considered wrappers, plastic bags, paper, food, wood scraps, etc…Dust is not considered garbage.* | **Answer = [ ]** | | | |
|  | **Answer in English** | **Answer Code** | | | **Skip To** |
|  | Yes | **1** | | | ***Q 57.0*** |
|  | No | **0** | | | ***If NO, complete Q 56.0 SPECIFY*** |
| **Q 56.0 Specify** | *Specify* | **________________________________________** | | | |
| **Q 57.0** | **Is compound floor clean?** | **Answer = [ ]** | | | |
|  | **Answer in English** | **Answer Code** | | | **Skip To** |
|  | Yes | **1** | | | ***Q 58.0*** |
|  | No | **0** | | | ***If NO, complete Q 57.0 SPECIFY*** |
| **Q 57.0 Specify** | *Specify* | **________________________________________** | | | |

| **Q 58.0** | **Are human feces visibleinside the household or compound?** (ask specifically what kind if feces are observed to verify) | | **Answer = [ ]** | | | |
| --- | --- | --- | --- | --- | --- | --- |
|  | **Answer in English** | | **Answer Code** | | **Skip To** | |
|  | Yes | | **1** | | **N/A** | |
|  | No | | **0** | |  |  |
| **Q 59.0** | **Is there a presence of flies in the household cooking area during the day?** | **Answer = [ ]** | | | |  |
|  | **Answer in English** | **Answer Code** | | **Skip To** | |  |
|  | Yes | **1** | | **N/A** | |  |
|  | No | **0** | |  |  |  |

| **OBSERVATIONS FOR SECTION 6** | | | |
| --- | --- | --- | --- |
| **Q 60.0** | **If plates and utensils have been cleaned after the last meal and are ready to be used for the next meal, do plates and utensils appear clean?** | **Answer = [ ]** | |
|  | **Answer in English** | **Answer Code** | **Skip To** |
|  | Yes  *(no spot of food or dirt anywhere on any plate or utensil!)* | **1** | ***Q 61.0*** |
|  | No | **0** | ***If NO, complete Q 60.0 SPECIFY*** |
|  | Unknown | **999** | **N/A** |
| **Q60.0 *Specify*** |  | | |

| **Q 61.0** | | **Adults and children aged 5 and over present in the household. *Please check back to demographics section for ID number*** | | | |
| --- | --- | --- | --- | --- | --- |
| **Q 61.1**  **Person ID Number** | **Q 61.2 Are the person’s hands clean (no visible dirt, no black fingernails)**  **1 = Yes**  **0 = No, specify**  **999 = Unknown** | | | **Q 61.3 Is person’s sari/clothing clean? (no visible fresh dirt/food/grime)**  **1= Yes (old stains like turmeric are still considered clean)**  **0= No, specify**  **999 = Unknown** | |
|  |  | | **Specify:**_______________________________ |  | **Specify:**_____________________________________ |
|  |  | | **Specify:**_______________________________ |  | **Specify:**_____________________________________ |
|  |  | | **Specify:**_______________________________ |  | **Specify:**_____________________________________ |
|  |  | | **Specify:**_______________________________ |  | **Specify:**_____________________________________ |
|  |  | | **Specify:**_______________________________ |  | **Specify:**_____________________________________ |
|  |  | | **Specify:**_______________________________ |  | **Specify:**_____________________________________ |
|  |  | | **Specify:**_______________________________ |  | **Specify:**_____________________________________ |
| **Q 62.0** | **Children under 5 present in the household. *Please check back to demographics section for ID number*.**  **If no children under 5 years old present in the household, then skip to *Q 63.0*.** | | | | |
| **Q 62.1 Person ID Number** | **Q 62.2 Are the person’s hands clean (no visible dirt, no black fingernails)**  **1= Yes**  **0= No, *specify***  **999= Unknown** | | | **Q 62.3 Is person’s sari/clothing clean? (no visible fresh dirt/food/ grime)**  **1= Yes (old stains like turmeric are still considered clean)**  **0= No, *specify***  **999 = Unknown** | |
|  |  | | **Specify:**__________________________________ |  | **Specify:**_____________________________________ |
|  |  | | **Specify:**__________________________________ |  | **Specify:**_____________________________________ |
|  |  | | **Specify:**__________________________________ |  | **Specify:**_____________________________________ |
|  |  | | **Specify:**__________________________________ |  | **Specify:**_____________________________________ |
|  |  | | **Specify:**__________________________________ |  | **Specify:**_____________________________________ |
|  |  | | **Specify:**__________________________________ |  | **Specify:**_____________________________________ |
|  |  | | **Specify:**__________________________________ |  | **Specify:**______________________________________ |

| **Q 63.0** | **Are there animals present in compound?** | **Answer = [ ]** | |
| --- | --- | --- | --- |
|  | **Answer in English** | **Answer code** | **Skip To** |
|  | Yes | **1** | **N/A** |
|  | No | **0** | ***Picture Section,***  ***Q 67.1, 2, 3...*** |
|  | Unknown | **999** | **N/A** |
| **Q 64.0** | **If yes, which types of animals?** *Multiple answers possible* | | |
| **Q 64.1** | **Answer in English** | **Y/N/O=1/0/777** | **Skip To** |
| **Q 64.2** | Goat |  | **N/A** |
| **Q 64.3** | Chicken |  |  |
| **Q 64.4** | Goose |  |  |
| **Q 64.5** | Dog |  |  |
| **Q 64.6** | Cat |  |  |
| **Q 64.7** | Duck |  |  |
| **Q 64.8** | Pet Birds |  |  |
| **Q 64.9** | Cow |  |  |
| **Q 64.10** | Mongoose |  |  |
| **Q 64.11** | Rat |  |  |
| **Q 64.12** | Other |  | ***If NO, skip to Q66.0*** |
| **Q 64.12 Other** | Other specify | **________________________________________** | |
| **Q 66.0** | **Are animals ranging freely in the household or compound?** | **Answer = [ ]** | |
|  | **Answer in English** | **Answer Code** | **Skip To** |
|  | Yes | **1** | **N/A** |
|  | No | **0** | ***Picture Section,***  ***Q 67.1, 2, 3...*** |
|  | Unknown | **999** | **N/A** |
| **Q 66.0 Specify** | *Specify* | **________________________________________** | |

| **PICTURES TO BE TAKEN** | | |
| --- | --- | --- |
| **Q 67.1** | Every room in household.  Picture Numbers |  |
|  |  |  |
|  |  |  |
|  |  |  |
| **Q 67.2** | Every latrine used by household.  Picture Numbers |  |
|  |  |  |
|  |  |  |
|  |  |  |
| **Q 67.3** | Every water source used by household. Picture Numbers |  |
|  |  |  |
|  |  |  |
|  |  |  |
| **Q 67.4** | All water storage containers.  Picture Numbers |  |
|  |  |  |
|  |  |  |
|  |  |  |
| **Q 67.5** | Cooking site.  Picture Numbers |  |
|  |  |  |
|  |  |  |
|  |  |  |
| **Q 68.0** | **End Time (00:00)** | ☐☐ :☐☐ |

**Signature of Interviewer (FRA) Date (DD.MM.YYYY) _________________________**

**Checked By (SFRO Signature) Date (DD.MM.YYYY)_________________________**
