# Supplementary material for: Water usage, hygiene and diarrhea in low-income urban communities—A mixed method prospective longitudinal study
Source: MethodsX. 2019 Nov 19;6:2822–37. doi: 10.1016/j.mex.2019.11.018 (PMC6909126; doi:10.1016/j.mex.2019.11.018)
Supplement: Supplementary file 4 [file mmc4.docx]

##

## Annex 2: Monthly visit questionnaire

| Q.a | FRA ID |
| --- | --- |
| Q.b | Date of survey (DD/Month/Year) |
| Q.c | Household ID |
| Q.c1 | Please re-enter the household ID |
| Q.d | Random listing number |
| Q.e | Which Survey Round is this? |
| Q.f | Is this the first Monthly visit to the household? (1=Yes, 0=No) |
| Q.f1 | Visit number to the household. |
| Q.g | Start |
| Q.h | Start time (HH : MIN) [Enter the survey start time] |

| NOTE: All text in **bold** should be read, and all text in *italics* are notes to the interviewer | | | | | | | | | | | | | | | | | | | |
| --- | --- | --- | --- | --- | --- | --- | --- | --- | --- | --- | --- | --- | --- | --- | --- | --- | --- | --- | --- |
| Section 1: Water use | | | | | | | | | | | | | | | | | | | |
| **Q.0.From how many different water points or sources did you collect water for your household since yesterday?**  (Recod the each water collection points details starting with the most water was collected.)  Skip Note: Enter 0, if there was no water points visited yesterday and skip to Q.7 | | | | | | | | | | | | | | | | | | | |
| **1. Type of water collection points from where you collected water yesterday?** *Note to FRA: Ask specifically about rain, pond, and river water on the first visit and also during rainy season*.  1=Tap  2=Pipe  3= Hand pump vertical pressure (tubewell)  4=Hand pump horizontal pressure (tubewell)  5=Well (with bucket)  6=River  7=Rainwater  8=Pond  777=Other, please specify……. | | | | | **2. Were you using this collection point the last time we visited? (**If this is the first round visit, please answer ‘No’)  1=Yes  0=No  999=DK  (*if yes, skip to Q5)* | | | | **3. From where water comes to this point/source of the water? (surface, WASA, ground, etc.)** 1=WASA supply  2=Individual submersible pump  3=Communal submersible pump  4=Well  (<100 ft)  5=Shallow tubewell (100-250ft)  6= Deep tubewell (>250ft)  7=Compressor pump (>100,<250ft)  777= Others please specify…….  999=*Unknown* | | | | | **4. What was the type of tank available for storage of household water?**  0=No tank  1=Roof tank  2=Ground tank with attached tap  4=Ground tank without attached tap 3=In-ground tank that pumps into roof tank  5=In-ground tank with Bucket  6=In-ground tank with tubewell 777=Other, please specify……. | | | **5. Is there more or less water comes or available from this collection point compared to the last we visited your household?**  1=More  2=Less  3=Same  888= N/A 999=*Unknown* | | **6. When was water flowing from the collection point since last 24 hour’s?**(If available 24 hours, write 00:00-23:59. A pond/ river/well would also be 00:00-23:59. And put 00:00-00:00 for 2nd &3rd time.  If no water was flowing, write 00:00-00:00)  From  ____ to ____ and  _____ to _____  999=*Unknown* |
| *S1* | |  | | *Other* |  | | | |  | *Other* | | | |  | | *Other* |  | | From ____ to ____ and  _____ to _____ |
| *S2* | |  | | *Other* |  | | | |  | *Other* | | | |  | | *Other* |  | | From ____ to ____ and  _____ to _____ |
| *S3* | |  | | *Other* |  | | | |  | *Other* | | | |  | | *Other* |  | | From ____ to ____ and  _____ to _____ |
| *S4* | |  | | *Other* |  | | | |  | *Other* | | | |  | | *Other* |  | | From ____ to ____ and  _____ to _____ |
| *S5* | |  | | *Other* |  | | | |  | *Other* | | | |  | | *Other* |  | | From ____ to ____ and  _____ to _____ |
| **Now I am going to ask you a series of questions about how you use the sources of water in your household.** | | | | | | | | | | | | | | | | | |  |  |
| **7a. Today is your drinking water clear or cloudy/dirty?** | | | | | | | | | | | | | | | | | |  |  |
| *Answer in English* | | | | | | | *Answer code* | | | | | | *Skip to* | | | | |  |  |
| Drinking water is clear | | | | | | | **1** | | | | | | N/A | | | | |  |  |
| Drinking water is cloudy, colored or dirty | | | | | | | **2** | | | | | | N/A | | | | |  |  |
| **7b. Today does your drinking water smells good or bad /strange?** | | | | | | |  | | | | | |  | | | | |  |  |
| *Answer in English* | | | | | | | *Answer code* | | | | | | *Skip to* | | | | |  |  |
| Water has a good smell | | | | | | | **1** | | | | | | N/A | | | | |  |  |
| Water has a bad smell | | | | | | | **2** | | | | | | N/A | | | | |  |  |
| **7c. Today how was the taste of your drinking water?** | | | | | | |  | | | | | |  | | | | |  |  |
| *Answer in English* | | | | | | | *Answer code* | | | | | | *Skip to* | | | | |  |  |
| The taste was better | | | | | | | **1** | | | | | | N/A | | | | |  |  |
| The taste was not good | | | | | | | **2** | | | | | | N/A | | | | |  |  |
| No taste was in it, seems it was okay to drink | | | | | | | **3** | | | | | | N/A | | | | |  |  |
| **8. Did you treat your drinking water today? If so, how?** | | | | | | | **Answer [ ]** | | | | | | | | | | |  |  |
| *Answer in English* | | | | | | | *Answer Code* | | | | | *Skip to* | | | | | |  |  |
| *Did not treat water* | | | | | | | *0* | | | | | N/A | | | | | |  |  |
| Boiling | | | | | | 1 | | | | | | N/A | | | | | |  |  |
| Alum | | | | | | 2 | | | | | | N/A | | | | | |  |  |
| Chlorine | | | | | | 3 | | | | | | N/A | | | | | |  |  |
| Purifying filter | | | | | | 4 | | | | | | N/A | | | | | |  |  |
| Boil and purifying filter | | | | | | 5 | | | | | | N/A | | | | | |  |  |
| Other | | | | | | 777 | | | | | | Specify | | | | | |  |  |
| *Other specify* | | | | | |  | | | | | | N/A | | | | | |  |  |
| *Unknown/DK* | | | | | | 999 | | | | | | N/A | | | | | |  |  |
| **9. Are you using more or less water overall in your daily life compared to our last visit at your household? (**Not applicable for first visit) | | | | | | **Answer [ ]** | | | | | | | | | | | |  |  |
| *Answer in English* | | | | | | *Answer code* | | | | | *Skip to* | | | | | | |  |  |
| More | | | | | | 1 | | | | | N/A | | | | | | |  |  |
| Less | | | | | | 0 | | | | | N/A | | | | | | |  |  |
| Same | | | | | | 2 | | | | | N/A | | | | | | |  |  |
| Not applicable for first visit | | | | | | 888 | | | | | N/A | | | | | | |  |  |
| *Unknown* | | | | | | 999 | | | | | N/A | | | | | | |  |  |
| **Section 2: Changes of water storage containers at households** | | | | | | | | | | | | | | | | | |  |  |
| **Now I am going to ask you some questions about where you store your water.** | | | | | | | | | | | | | | | | | |  |  |
| **10.** | **Other than the containers you have shown us during our last visit how many additional containers your household is using now for water storage?** | | | | | | | Answer [ ]  Enter 0, if the household didn’t used any new large containers. And skip to Q.12 | | | | | | | | | |  |  |
| **Could you please show me the containers those we did not see during our last visit?**  *Note to FRA: please verify if this is a carrier container so that it can be incorporated in the water quantification sheet.* | | | | | | | | | | | | | | | | | |  |  |
| **11a.Type of this new container**  *1= Plastic bucket*  *2=Aluminum/silver bucket*  *3=Aluminum/silverKolshi*  *4=Claykolshi*  *5=Plastic drum*  *6=Plastic bottle*  *7=Plastic Jug*  *8=Plastic Jerrycan*  *9=Glass bottle/jug*  *777= Other, specify…* | | | | | | | | | | | | | | | 11.b **Size in liters of this new container** | | |  |  |
|  | | | *Other:* | | | | | | | | | | | | Liters | | |  |  |
|  | | | *Other:* | | | | | | | | | | | | Liters | | |  |  |
|  | | | *Other:* | | | | | | | | | | | | Liters | | |  |  |
| 11.c Insert an ID for this new container such as CC1, CC2, CC3… etc. | | | | | | | | | | | | | | |  | | |  |  |

| **12.** | **How many storage containers (for those we inserted container number during our other visits) have you thrown away since the last time we visited you?** | Answer [ ]  Enter 0, if the household did not discard any containers. And skip to Q.12.b | | |
| --- | --- | --- | --- | --- |
| **12a** | **What was the container ID that you have stopped using since our last visit?** | Answer [ ]  Enter ID # for each of the container that reported in Q.12 | | |
|  | *Answer in English* | *Answer code* | *Skip to* | |
|  | **Container ID_________** | C1, C2, C3… | N/A | |
|  | **Container ID_________** | C1, C2, C3… | N/A | |
|  | **Container ID_________** | C1, C2, C3… | N/A | |
|  | *Unknown* | 999 | N/A | |
|  | *If the respondent cannot remember, show them the saved picture of all of their household containers in the note at the bottom of the page.* | | | |
| **12b** | If there are new water containers uses the household or discarded any water containers since the last visiting of the household, please take picture for all of them and label the picture as- 001. 16.11.2014 | | | [picture] |

| **Section 3: Changes in household members** | | | | | | | | | | | | | | |  |  |  |
| --- | --- | --- | --- | --- | --- | --- | --- | --- | --- | --- | --- | --- | --- | --- | --- | --- | --- |
| **13.a** | **How many people are currently visiting and intended to stay <6 months in the household?** (*This also includes a new child*) | | | | Answer [ ]  Enter 0, if there are no visitors currently staing in the household. | | | | | | | | | |  |  |  |
| **13.b** | Since our last visit, h**ow many new people are living in the household? (intend to stay >6 months)** (*This also includes a new child*) | | | | Answer [ ]  Enter 0, if there are no new people living in the household. And skip to Q.14a  Either continues the ques. 13b1-13b4 for each of the reported number. | | | | | | | | | |  |  |  |
| **13b1** | **What is the name of new person living in the household?** | | | |  | | | | | | | | | |  |  |  |
| **13b2** | **How old the new person living in the household?** | | | | *_____________ Years* | | | | | | | | | |  |  |  |
| **13b3** | **The sex of the new person living in the household?** | | | |  | | | | |  | | | | |  |  |  |
|  | *Answer in English* | | | | *Answer code* | | | | | *Skip to* | | | | |  |  |  |
|  | **Male** | | | | 1 | | | | | N/A | | | | |  |  |  |
|  | **Female** | | | | 0 | | | | | N/A | | | | |  |  |  |
| **13b4** | **Record a new Person ID for that people:**  *Also, please report this to the field manager so that it can be updated for the household visits* | | | | ☐☐  ☐☐  ☐☐ | | | | | | | | | |  |  |  |
|  | Note: After the interview, please cosent all the new people LIVING in the household to participate in this study. | | | |  | | | | | | | | | |  |  |  |
| **14a** | **How many household members are absent since the last time we visited your household?** | | | | Answer [ ] | | | | | | | | | |  |  |  |
| **14b.** | **How many people have permanently moved out of the household since the last time we visited you?** (Moved out means they are not planning to return) | | | | Answer [ ]  Enter 0, if there are no person permanently moved out from the household **since the last monthly visit**. And skip to Section-4.  Either continues the ques. 14c for each of the reported number. | | | | | | | | | |  |  |  |
| **14c.** | **What was the person ID of permanently moved out people?**  *Also, please report this to the field manager so that it can be updated for the household visits* | | | | ☐☐  ☐☐  ☐☐ | | | | | N/A | | | | |  |  |  |
|  | | | | | | | | | | | | | | |  |  |  |
| Section 4: food handling at household | | | | | | | | | | | | | | |  |  |  |
| **Now I am going to ask you a series of questions about how you handle food in your household and what you eat.** | | | | | | | | | | | | | | |  |  |  |
| **15. Which of these items did your household member/s eat yesterday at home in different time period (Breakfast, Lunch, Dinner & Snacks)?** | | | | | | | | | | | | | | |  |  |  |
| *Name of the foods in English* | | | **15a.Breakfast**  Y/N=1/0 | | | | **15b.Lunch**  Y/N=1/0 | | **15c.Dinner**  Y/N=1/0 | | | **15d.Snacks**  Y/N=1/0 | | |  |  |  |
| *Answer in English* | | | *Answer code* | | | | *Answer code* | | *Answer code* | | | *Answer code* | | |  |  |  |
| **1. Rice** (rice, pulao, kitchuri, biryani,noodles, rice pudding, shuji,etc.) | | |  | | | |  | |  | | |  | | |  |  |  |
| 2.Rice soaked in water (pantha, chira, muri, ruti, chatu, khai etc. *where drinking water was added*) | | |  | | | |  | |  | | |  | | |  |  |  |
| **3.Bread (biscuits, shingara, roti, chapatti, naan, chips, cake, etc.)** | | |  | | | |  | |  | | |  | | |  |  |  |
| **4. Meat (beef, mutton or chicken)** | | |  | | | |  | |  | | |  | | |  |  |  |
| **5. Fish/dried fish** | | |  | | | |  | |  | | |  | | |  |  |  |
| **6. Curry** | | |  | | | |  | |  | | |  | | |  |  |  |
| **7. Cooked Vegetables (any vegetables, potato or eggplant)** | | |  | | | |  | |  | | |  | | |  |  |  |
| **8. Raw Vegetables(cucumber, carrot, tomato, onion, chili, mula, etc.)** | | |  | | | |  | |  | | |  | | |  |  |  |
| **9. Eggs** | | |  | | | |  | |  | | |  | | |  |  |  |
| **10. Lentils** | | |  | | | |  | |  | | |  | | |  |  |  |
| **11. Milk** | | |  | | | |  | |  | | |  | | |  |  |  |
| **12. Fruit** | | |  | | | |  | |  | | |  | | |  |  |  |
| **13. Betel leaf and nut (paansupari)** | | |  | | | |  | |  | | |  | | |  |  |  |
| **14.** Baby food (other than those not mentioned above) | | |  | | | |  | |  | | |  | | |  |  |  |
| **15. Pickles** | | |  | | | |  | |  | | |  | | |  |  |  |
| **99** Do not remember | | |  | | | |  | |  | | |  | | |  |  |  |
| **77.Others.......................** | | |  | | | |  | |  | | |  | | |  |  |  |
| **Other specify** | | |  | | | |  | |  | | |  | | |  |  |  |
| **88.** | | |  | | | |  | |  | | |  | | |  |  |  |
|  | | | | | | | | | | | | | |  |  |  |  |
| **16.** | | **How many times did you cook yesterday for your household?** | | | | Answer [ ] | | | | | | | | |  |  |  |
|  | | *Answer in English* | | | | *Answer code* | | | | | *Skip to* | | | |  |  |  |
|  | | I did not cook | | | | 0 | | | | | N/A | | | |  |  |  |
|  | | 1time | | | | 1 | | | | | N/A | | | |  |  |  |
|  | | 2 times | | | | 2 | | | | | N/A | | | |  |  |  |
|  | | 3 times | | | | 3 | | | | | N/A | | | |  |  |  |
|  | | 4 times/or more | | | | 4 | | | | | N/A | | | |  |  |  |
| **17.** | | **How many types of food you have today that you cooked and stored yesterday and will eat/ have eaten today?** | | | | Answer [ ]  Enter 0, if they have no food stored from yesterday. And skip to Section-5.  Either continues the ques. 18-18.4 for each of the reported number/item. | | | | | | | | |  |  |  |
| **18 The type of food you stored yesterday,**  1. Rice (rice, pulao, kitchuri, biryani, noodles, rice pudding, shuji, etc.)  2. Rice soaked in water (pantha, chira, muri, ruti, chatu, khai etc. *where drinking water was added*)  3. Bread (biscuits, shingara, roti, chapatti, naan, chips, cake, etc.)  4. Meat (beef, mutton or chicken)  5. Fish/dried fish  6. Curry  7. Cooked Vegetables (any vegetables, potato or eggplant)  8. **Raw Vegetables(cucumber, carrot, tomato, onion, chili, mula, etc.)**  9. Eggs  10. Lentils  11.Milk  12.Fruits  13. **Betel leaf and nut (paansupari)**  14. Baby food (other than those not mentioned above)  **15. Pickles**  77. Others........  **99** Do not remember | | | | **18.1 Was the food stored in a refrigerator?**  0=no  1=yes  999= *Unknown* | | | | **18.2 Did you/Will you reheat the food before eating it today?**  0=no  1=yes  999= *Unknown* | | | | | **18.3 Was the stored food covered with a lid or gauze?**  0=no  1=yes  999= *Unknown* | | | **18.4 Was water added to the stored food before eating today?**  0=no  1=yes  999= *Unknown* | **18.5 If water was added to the stored food, did you reheat before eating?**  0=no  1=yes  999= *Unknown*  **888=NA** |
| Answer  [ ] | | | | Answer  [ ] | | | | Answer  [ ] | | | | | Answer  [ ] | | | Answer  [ ] | |
| Answer  [ ] | | | | Answer  [ ] | | | | Answer  [ ] | | | | | Answer  [ ] | | | Answer  [ ] | |
| Answer  [ ] | | | | Answer  [ ] | | | | Answer  [ ] | | | | | Answer  [ ] | | | Answer  [ ] | |
| Answer  [ ] | | | | Answer  [ ] | | | | Answer  [ ] | | | | | Answer  [ ] | | | Answer  [ ] | |
| Answer  [ ] | | | | Answer  [ ] | | | | Answer  [ ] | | | | | Answer  [ ] | | | Answer  [ ] | |

| **Section 5: Loose stools** | | | | | | | | | | |  |  |
| --- | --- | --- | --- | --- | --- | --- | --- | --- | --- | --- | --- | --- |
| **I am now going to ask you a few questions aboutyour health .** | | | | | | | | | | |  |  |
| **19.** | **In the past 2 days, how many people in your household had 1 or more loose stools in a 24 hour period?** | | **Answer [ ]**  Enter 0, if no one have diarrhea, 666 for refuse, 999 for DK and skip to Q.20.  Either continues the ques. 19a for each of the reported person. | | | | | | | |  |  |
| **19a** | Person’s ID of diarrhea affected people: | | ☐☐  ☐☐  ☐☐  ☐☐  ☐☐ | | | | | | | |  |  |
| **19b** | Did you call to choleraphone? | | Please ask this question if there was a symptom of diarrhea reported in Q.19, otherwise select Not Applicable. | | | | | | | |  |  |
|  | *Answer in English* | | *Answer code* | | | | | *Skip to* | | |  |  |
|  | Yes | | 1 | | | | | Q.20 | | |  |  |
|  | No | | 0 | | | | | NA | | |  |  |
|  | *DK/Unknown* | | 999 | | | | | Q.20 | | |  |  |
|  | Not Applicable | | 888 | | | | | Q.20 | | |  |  |
| **19c** | If did not call, then why? | |  | | | | | | | |  |  |
|  | *Answer in English* | | *Answer code* | | | | *Skip to* | | | |  |  |
|  | No phone was given | | 1 | | | |  | | | |  |  |
|  | Phone is not functioning | | 2 | | | |  | | | |  |  |
|  | Forgot or the available members at home do not know how to call | | 3 | | | |  | | | |  |  |
|  | Not interested to call | | 4 | | | |  | | | |  |  |
|  | Others (specify) | | 777 | | | |  | | | |  |  |
|  | Not Applicable | | 888 | | | | NA | | | |  |  |
| **20** | **Can I please see your choleraphone?** | | Answer [0=No, 1=yes ] | | | | | | | |  |  |
|  | Yes, *If yes, ask caretaker to call the Choleraphone hotline to check the number. Talk to the call center person to check the household mobile number.Pleaserecharge 100 taka to the phone using the scratch card* | | 1 | | | N/A | | | | |  |  |
|  | If *the Choleraphone* is not in the home. *They do not receive phone credit. Please explain to the caretaker that part of the terms of their enrollment is that they must keep the Choleraphone with them.When the phone is back with the caretaker, he/she may call to request that credit be given the following day.* | | 0 | | | N/A | | | | |  |  |
| \| **Section 6: Observations:**  **Complete observations in and around the household** \| \| \| \| \| \| \| \| \| \| --- \| --- \| --- \| --- \| --- \| --- \| --- \| --- \| --- \| \| **21.** \| \| Are human feces visible anywhere in latrines (latrine floor, pan, or slab)? \| \| Answer [ ] \| \| \| \| \|  \| \| *Answer in English* \| \| *Answer code* \| \| *Skip to* \| \| \|  \| \| Yes \| \| 1 \| \| N/A \| \| \|  \| \| No \| \| 0 \| \| N/A \| \| \|  \| \| *DK/Unknown* \| \| 999 \| \| N/A \| \| \| *For questions 22-25, the corridor floor is always considered compound floor* \| \| \| \| \| \| \| \| \| \| **22.** \| Are human feces visible anywhere in the household or the courtyard? \| \| Answer [ ] \| \| \| \| \|  \| *Answer in English* \| \| *Answer code* \| \| *Skip to* \| \| \|  \| Yes \| \| 1 \| \| N/A \| \| \|  \| No \| \| 0 \| \| N/A \| \| \|  \| *Unknown* \| \| 999 \| \| N/A \| \| \| **23.** \| Are human feces visible in drains within the compound? \| \| Answer [ ] \| \| \| \| \|  \| *Answer in English* \| \| *Answer code* \| \| *Skip to* \| \| \|  \| Yes \| \| 1 \| \| N/A \| \| \|  \| No \| \| 0 \| \| N/A \| \| \|  \| Not Applicable \| \| 888 \| \| N/A \| \| \|  \| *Unknown* \| \| 999 \| \| N/A \| \| | | | | | | | | | | | | |
|  |  |  |  |  |  |  |  |  |  |  |  |  |
| \| **24.** \| Is the household floor clean? *(i.e. there was no garbage or wastes lying over the flor of the household )* \| Answer [ ] \| \| \| --- \| --- \| --- \| --- \| \|  \| *Answer in English* \| *Answer code* \| *Skip to* \| \|  \| Yes \| 1 \| N/A \| \|  \| No \| 0 \| N/A \| \|  \| *Unknown* \| 999 \| N/A \|  \| **25.** \| Is compound floor clean? ( *i.e there was no household garbage or wastages lying inside the compound anywhere)* \| Answer [ ] \| \| \| --- \| --- \| --- \| --- \| \|  \| *Answer in English* \| *Answer code* \| *Skip to* \| \|  \| Yes \| 1 \| N/A \| \|  \| No \| 0 \| N/A \| \|  \| *Unknown* \| 999 \| N/A \|   **Section 7: Water use quantification:** | | | | | | | | | | | |  |
| Measurements for water storage containers used since last 24 hours. To be taken from tally sheet. | | | | | | | | | | | |  |
| **26.How many water storage containers did your household use** since last 24 hours**?** | | | | | | | | | | | |  |
| 26.a ContainerID (Enter 888 for NA & 999 for DK) | | 26.b Container size in Liters | | | | | | | 26.c Number of times was filled the container since last 24 hours? | | |  |
|  | |  | | | | | | | ☐☐ | | |  |
|  | |  | | | | | | | ☐☐ | | |  |
|  | |  | | | | | | | ☐☐ | | |  |
| **27. Were there any containers filled at the water collection point during the last 24 hours that are not included on your water quantification sheet?**  If yes, please record a new container ID in question 26, the container size and how many times it was filled.  Then take a picture of the container and give it to the field manager so he can have it added to a new water quantification sheet for the household. | | | | | | | | | | | |  |
| **28.** Were any water activities done without a container since last 24 hours? | | Answer [ ] | | | | | | | |  |  |  |
| *Answer in English* | | *Answer code* | | | *Skip to* | | | | |  |  |  |
| Yes | | 1 | | | N/A | | | | |  |  |  |
| No | | 0 | | | End time | | | | |  |  |  |
| DK | | 999 | | | End time | | | | |  |  |  |
| *How many times was each of the following activities done WITHIN the compound without using any container* since last 24 hours*?* | | | | | | | | | |  |  |  |
| **Activities** | | | | **How many times/numbers** | | | | | |  |  |  |
| **28a**. Washing hands (include all members in the household) | | | | ☐☐ | | | | | |  |  |  |
| **28b**. Bathing (an adult or child>5years) | | | | ☐☐ | | | | | |  |  |  |
| **28c**. Bathing ( children<5years old) | | | | ☐☐ | | | | | |  |  |  |
| **28d**. Washing plates/utensils | | | | ☐☐ | | | | | |  |  |  |
| **28e**. Washing clothes | | | | ☐☐ | | | | | |  |  |  |

| **29.** *How many times was each of the following activities done OUTSIDE the compound without using any container* since last 24 hours*?* | | |  |
| --- | --- | --- | --- |
| **Activities** | | **How many times/numbers** |  |
| **29a**. Washing hands (include all members in the household) | | ☐☐ |  |
| **29b**. Bathing (an adult or child>5years) | | ☐☐ |  |
| **29c**. Bathing ( children<5years old) | | ☐☐ |  |
| **29d**. Washing plates/utensils | | ☐☐ |  |
| **29e**. Washing clothes | | ☐☐ |  |
| End time of interview: | _____:_____ HH : MIN | | |
| FRA name |  | | |

____________________ ____________________

Signature of Interviewer (FRA) & Date Checked by (FRO Signature & Date)
