# Supplementary material for: Water usage, hygiene and diarrhea in low-income urban communities—A mixed method prospective longitudinal study
Source: MethodsX. 2019 Nov 19;6:2822–37. doi: 10.1016/j.mex.2019.11.018 (PMC6909126; doi:10.1016/j.mex.2019.11.018)
Supplement: Supplementary file 5 [file mmc5.docx]

##

## Annex 4: Household questionnaire for diarrhea affected person

NOTE: Please fill up the questionnaire for every person of sampled household when someone affected with diarrhea and reported

NOTE: All text in bold should be read and all text in *italics* are notes to the interviewer.

(To be administered within 48 hours after receiving cholera phone call)

Now I am going to ask you some questions:

Section 1: Questionnaire identification

a. Questionnaire Id .....................................................................

b. HH Id.................................................................................................................

c. Person Id ................................................................................................................

d. Visit Number ..................................................................................................................

d.1. Random listing number..............................................................

e. Date of interview: DD/Month/Year ................... //

f. Start time of interview: HH : Min ............................... :

g. Who was the patient?

1= Child (0-10 years)

2= 11-17 years aged child

3= 17+ years

g.1. Sex of the patient?

1=Male

2= Female

h. Respondent type ................................................................................................................

Self (Patient ) = 1

Other than patient = 2

**For children :**

Mother =3, Father =4 Grant Mother =5, Grant Father =6, Sister =7, Brother =8, Uncle=9, Aunty =10, Other, specify =777

i. How the diarrheal information for this household/person was found? ......................

Code :

Through cholera phone =1,

During monthly visits = 2,

Through follow up phone call = 3, Through random visit = 4,

Others, specify =777

j. Name & employee number of data collector ...............................

| Section 2: Diarrhoea | | | | | | | | | |
| --- | --- | --- | --- | --- | --- | --- | --- | --- | --- |
| Introducing question |  | | Answer =[ ] | | | | | |  |
| Q.1 | How many loose stools have you had in the last 24 hours? | |  | | | | | |  |
|  | *Answer in English* | | *Answer code* | | *Skip to* | | | |  |
|  | None | | 0 | | N/A | | | |  |
|  | 1time | | 1 | | N/A | | | |  |
|  | 2 times | | 2 | | N/A | | | |  |
|  | 3 times | | 3 | | N/A | | | |  |
|  | 3 times 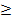 | | 4 | | N/A | | | |  |
|  | Decline | | 666 | | N/A | | | |  |
| Q.2 | How many days ago did you start having loose stools? | | Answer =[ ] | | | | | |  |
|  | *Answer in English* | | *Answer code* | | | | *Skip to* | |  |
|  | Today | | 1 | | | | N/A | |  |
|  | Yesterday | | 2 | | | | N/A | |  |
|  | 2 days ago | | 3 | | | | N/A | |  |
|  | 3 or more days ago | | 4 | | | | N/A | |  |
|  | *Unknown* | | 999 | | | | N/A | |  |
|  | *Decline* | | 666 | | | | N/A | |  |
| Q.3 | | At what time of the day did you start having loose stools? | Answer =[ ] | | | | | |  |
|  | | *Answer in English* | *Answer code* | | | | *Skip to* | |  |
|  | | Morning | 1 | | | | N/A | |  |
|  | | Noon | 2 | | | | N/A | |  |
|  | | Evening | 3 | | | | N/A | |  |
|  | | Night | 4 | | | | N/A | |  |
|  | | *Unknown* | 999 | | | | N/A | |  |
|  | | *Decline* | 666 | | | | N/A | |  |
| Q.4 | | Did you / your child have any loose stools since you called? | | Answer =[ ] | | | |  |  |
|  | | *Answer in English* | | *Answer code* | | *Skip to* | |  |  |
|  | | Yes | | 1 | | N/A | |  |  |
|  | | No | | 0 | | N/A | |  |  |
|  | | Unknown | | 999 | | N/A | |  |  |
|  | | *Decline* | | 666 | | N/A | |  |  |
| Q.5 | | Approximately how many times loose stools have you/ your child passed untill now: | | Answer =[ ] | | | |  |  |
|  | | *Answer in English* | | *Answer code* | | *Skip to* | |  |  |
|  | | Insert the actual number of times | |  | | N/A | |  |  |
|  | | 15+ | | 8888 | |  | |  |  |
|  | | *Decline* | | 666 | | N/A | |  |  |
| Q.6 | | Since you/ your child started loose stools were any stools colorless? | | Answer =[ ] | | | |  |  |
|  | | *Answer in English* | | *Answer code* | | *Skip to* | |  |  |
|  | | Yes | | 1 | | N/A | |  |  |
|  | | No | | 0 | | N/A | |  |  |
|  | | *Unknown* | | 999 | | N/A | |  |  |
|  | | *Decline* | | 666 | | N/A | |  |  |
| Q.7 | | Since you started having loose stools were any stools water-like? | | Answer =[ ] | | | |  |  |
|  | | *Answer in English* | | *Answer code* | | *Skip to* | |  |  |
|  | | Yes | | 1 | | N/A | |  |  |
|  | | No | | 0 | | N/A | |  |  |
|  | | *Unknown* | | 999 | | N/A | |  |  |
|  | | *Decline* | | 666 | | N/A | |  |  |
| Q.8 | | Since the diarrhea started were any stools passes like bellow? (Should be allowed multiple answer) | | | | | |  |  |
|  | | *Answer in English* | | *Answer code* | | *Skip to* | |  |  |
| Q.8.1 | | With blood | | Y/N=1/0 | | N/A | |  |  |
| Q.8.2 | | Black | | Y/N=1/0 | | N/A | |  |  |
| Q.8.3 | | With mucous | | Y/N=1/0 | | N/A | |  |  |
| Q.8.4 | | None of the above | | Y/N=1/0 | |  | |  |  |
| Q.8.99 | | *Unknown* | | Y/N=1/0 | |  | |  |  |
| Q.8.66 | | *Refused/decline* | | Y/N=1/0 | |  | |  |  |
| Q.9 | | Since the diarrhea started, did you /your child experience any of the following? ( Should be allowed multiple answer) | | | | | |  |  |
|  | | *Answer in English* | | *Answer code* | | *Skip to* | |  |  |
| Q.9.1 | | Fever | | Y/N=1/0 | | N/A | |  |  |
| Q.9.2 | | Vomiting | | Y/N=1/0 | | N/A | |  |  |
| Q.9.3 | | Being tired | | Y/N=1/0 | | N/A | |  |  |
| Q.9.4 | | Fainting | | Y/N=1/0 | | N/A | |  |  |
| Q.9.5 | | Cramping | | Y/N=1/0 | | N/A | |  |  |
| Q.9.6 | | None of the above | | Y/N=1/0 | |  | |  |  |
| Q.9.99 | | *Unknown* | | Y/N=1/0 | | N/A | |  |  |
| Q.10 | | How many times if any have you /your child visited a community clinic/hospital/ pharmacy in response to this episode of loose stools? | | Answer =[ ] | | | |  |  |
|  | | *Answer in English* | | *Answer code* | | *Skip to* | |  |  |
|  | | None | | 0 | | Q.12 | |  |  |
|  | | _____Times | | 1/2/…….. | | N/A | |  |  |
|  | | *Decline* | | 666 | | N/A | |  |  |
| Q.11 | | Type of health facilities you/ your child visited to get treatment for this episode of loose stools? (Should be allowed multiple answer) | | Answer =[ ] | | | |  |  |
|  | | *Answer in English* | | *Answer code* | | *Skip to* | |  |  |
| Q.11.1 | | Community clinic | | Y/N=1/0 | | N/A | |  |  |
| Q.11.2 | | Govt. Hospital | | Y/N=1/0 | | N/A | |  |  |
| Q.11.3 | | ICCDR,B/The Cholera Hospital | | Y/N=1/0 | | N/A | |  |  |
| Q.11.4 | | Private /NGO Clinic/Hospital | | Y/N=1/0 | | N/A | |  |  |
| Q.11.5 | | Private Practitioners (physician/doctor) | | Y/N=1/0 | | N/A | |  |  |
| Q.11.6 | | Pharmacy/Drug seller | | Y/N=1/0 | | N/A | |  |  |
| Q.11.7 | | Unqualified healthcare practitioners (quack doctor or spiritual or religious healers or health worker) | | Y/N=1/0 | | N/A | |  |  |
| Q.11.77 | | Other, specify______________ | | Y/N=1/0 | | N/A | |  |  |
| Q.12 | | Was any test/diagnosis done? | | Answer =[ ] | | | |  |  |
|  | | *Answer in English* | | *Answer code* | | *Skip to* | |  |  |
|  | | Yes, diagnosis was done _____ | | 1 | | N/A | |  |  |
|  | | DK/Do not remember | | 999 | | N/A | |  |  |
|  | | No test done | | 0 | | N/A | |  |  |
| Q12.a | | If Q12 was yes, what was the results? | | *Answer code* | | *Skip to* | |  |  |
|  | | Write the name of disease: __________________ | | *text* | | *N/A* | |  |  |
|  | | Result do not know/forgotten | | 999 | | N/A | |  |  |
|  | | Not applicable | | 888 | | N/A | |  |  |
| Q.13 | | Have you/your child taken any medication or ORS for the loose stools? | | Answer =[ ] | | | |  |  |
|  | | *Answer in English* | | *Answer code* | | *Skip to* | |  |  |
|  | | Yes | | 1 | | N/A | |  |  |
|  | | No | | 0 | | Q.15 | |  |  |
|  | | *Decline* | | 666 | | Q.15 | |  |  |
| Q.14 | | What type of medication have you /your child taken? (Should be allowed multiple answer) | | Answer =[ ]  *multiple options possible* | | | |  |  |
|  | | *Answer in English* | | *Answer code* | | *Skip to* | |  |  |
| Q.14.1 | | ORS (Saline) from Pharmacy | | Y/N=1/0 | | N/A | |  |  |
| Q.14.2 | | Homemade ORS | | Y/N=1/0 | | N/A | |  |  |
| Q.14.3 | | Medicine | | Y/N=1/0 | | N/A | |  |  |
| Q.14.3.a | | If medicine taken, please specify the name of medicine:__________ | |  | |  | |  |  |
| Q.14.4 | | Intravenous rehydration | | Y/N=1/0 | | N/A | |  |  |
| Q.14.5 | | Herbal medicine | | Y/N=1/0 | | N/A | |  |  |
| Q.14.6 | | Chanting water | | Y/N=1/0 | | N/A | |  |  |
| Q.14.77 | | Other specify______ | | Y/N=1/0 | | N/A | |  |  |
| Q.14.99 | | Forgotten/DK | | Y/N=1/0 | | N/A | |  |  |
| Q.15 | | Did the present episode of loose stools inhibit you from going to work/ schooling? | | Y/N/NA=1/0/888 | | N/A | |  |  |
|  | | *Answer in English* | | *Answer code* | | *Skip to* | |  |  |
|  | | Yes | | 1 | | N/A | |  |  |
|  | | No | | 0 | | Q.16 | |  |  |
|  | | Unknown | | 999 | |  | |  |  |
|  | | Decline | | 666 | | Q.16 | |  |  |
|  | | N/A | | 888 | |  | |  |  |
| Q.15.a | | If yes, specify how many days you did not go to work/ schooling. | | ------------days | | | |  |  |
| Q.15.b | | Follow up question in next 24 hours time prior to receiving the call from the household (If 1 or 2 episodes of loose stool passed till the time of house visit):  How many times loose stool passed altogether on completion 24 hours after the call from the household (ask over phone); | | Answer =[ ] | | | |  |  |
|  | | 1 | | 1 | | | |  |  |
|  | | 2 | | 2 | | | |  |  |
|  | | 3 | | 3 | | | |  |  |
|  | | More than 3 | | 4 | | | |  |  |
|  | | Not applicable  (already there was 3 or more episode of loose motion at the time of house visit) | | 888 | | | |  |  |

|  | Section 3: water use  I am going to ask you some questions about your/your child water use in the household. | | | | | | | |  |  |
| --- | --- | --- | --- | --- | --- | --- | --- | --- | --- | --- |
| Q.16 | | | Are you/your child using a different water source in your home since we interviewed you the last time? | Answer =[ ] | | | | |  |  |
|  | | | *Answer in English* | *Answer code* | | | *Skip to* | |  |  |
|  | | | Yes | 1 | | | N/A | |  |  |
|  | | | No | 0 | | | Q.18 | |  |  |
|  | | | *Unknown* | 999 | | | Q.18 | |  |  |
|  | | | *Decline* | 666 | | | Q.18 | |  |  |
| Q.16.a. | | | If you/your child are using another source water, can you show me where you would go to fill a glass of drinking water? | Answer =[ ] | | | | |  |  |
|  | | | *Answer in English* | *Answer code* | | | *Skip to* | |  |  |
|  | | | Tap | *1* | | | N/A | |  |  |
|  | | | Pipe | *2* | | |  | |  |  |
|  | | | Hand pump vertical pressure (tubewell) | *3* | | |  | |  |  |
|  | | | Hand pump horizontal pressure (tubewell) | *4* | | |  | |  |  |
|  | | | Well (with bucket) | 5 | | |  | |  |  |
|  | | | River | 6 | | |  | |  |  |
|  | | | Rainwater | 7 | | |  | |  |  |
|  | | | Pond | 8 | | |  | |  |  |
|  | | | Other, please specify | 777 | | |  | |  |  |
|  | | | N/A | 888 | | |  | |  |  |
|  | | | *unknown* | 999 | | |  | |  |  |
|  | | | *decline* | 666 | | |  | |  |  |
| Q.16.b. | | | The source of that water was - | Answer =[ ] | | | | |  |  |
|  | | | *Answer in English* | *Answer code* | | | *Skip to* | |  |  |
|  | | | WASA supply | 1 | | | N/A | |  |  |
|  | | | Individual submersible pump | 2 | | |  | |  |  |
|  | | | Communal submersible pump | 3 | | |  | |  |  |
|  | | | Well (<100 ft) | 4 | | |  | |  |  |
|  | | | Shallow tube-well (100-250ft) | 5 | | |  | |  |  |
|  | | | Deep tube-well (>250ft) | 6 | | |  | |  |  |
|  | | | Compressor pump (>100,<250ft) | 7 | | |  | |  |  |
|  | | | Other, please specify | 777 | | |  | |  |  |
|  | | | N/A | 888 | | |  | |  |  |
|  | | | Unknown | 999 | | |  | |  |  |
|  | | | Decline | 666 | | |  | |  |  |
| Q.16.c. | | | Type of tank the water was stored? | Answer =[ ] | | | | |  |  |
|  | | | *Answer in English* | *Answer code* | | | *Skip to* | |  |  |
|  | | | No tank | 0 | | |  | |  |  |
|  | | | Roof tank | 1 | | |  | |  |  |
|  | | | Ground tank with attached tap | 2 | | |  | |  |  |
|  | | | In-ground tank that pumps into roof-tank | 3 | | |  | |  |  |
|  | | | Ground tank without attached tap | 4 | | |  | |  |  |
|  | | | In-ground tank with Bucket | 5 | | |  | |  |  |
|  | | | In-ground tank with tube-well | 6 | | |  | |  |  |
|  | | | Other, please specify | 777 | | |  | |  |  |
|  | | | Unknown | 999 | | |  | |  |  |
|  | | | N/A | 888 | | |  | |  |  |
|  | | | Unknown | 999 | | |  | |  |  |
|  | | | Decline | 666 | | |  | |  |  |
| Q.16.d | | | *Why did you change the water source?* | Answer =[ ] | | | | |  |  |
|  | | | *Answer in English* | *Answer code* | | | *Skip to* | |  |  |
|  | | | Because of long distance to water source | 1 | | |  | |  |  |
|  | | | Because of bad quality of water source | 2 | | |  | |  |  |
|  | | | Because of low quantity in water source | 3 | | |  | |  |  |
|  | | | There was no water available in the source | 4 | | |  | |  |  |
|  | | | Other, please specify | 777 | | |  | |  |  |
|  | | | N/A | 888 | | |  | |  |  |
|  | | | Unknown | 999 | | |  | |  |  |
|  | | | Decline | 666 | | |  | |  |  |
| Q.17 | | For what the purpose you used the water that you brought from a different source. (Should be allowed multiple answer) | | Answer =[ ] | | | | |  |  |
|  | | *Answer in English* | | *Answer code* | | *Skip to* | | |  |  |
| Q.17.1 | | Drinking | | Y/N=1/0 | | N/A | | |  |  |
| Q.17.2 | | Cleaning the house | | Y/N=1/0 | | N/A | | |  |  |
| Q.17.3 | | Bathing children | | Y/N=1/0 | | N/A | | |  |  |
| Q.17.4 | | Bathing self | | Y/N=1/0 | | N/A | | |  |  |
| Q.17.5 | | Washing hands | | Y/N=1/0 | | N/A | | |  |  |
| Q.17.6 | | Washing plates/utensils | | Y/N=1/0 | | N/A | | |  |  |
| Q.17.7 | | Washing clothes | | Y/N=1/0 | | N/A | | |  |  |
| Q.17.8 | | Cooking | | Y/N=1/0 | | N/A | | |  |  |
| Q.17.9 | | Sanitation | | Y/N=1/0 | | N/A | | |  |  |
| Q.17.10 | | Adding water to leftover food | | Y/N=1/0 | | N/A | | |  |  |
| Q.17.11 | | Preparing tea | | Y/N=1/0 | | N/A | | |  |  |
| Q.17.12 | | Gargling | | Y/N=1/0 | | N/A | | |  |  |
| Q.17.13 | | Ablution | | Y/N=1/0 | | N/A | | |  |  |
| Q.17.77 | | Other | | Y/N=1/0 | | N/A | | |  |  |
| Q.17.99 | | Un-known | | Y/N=1/0 | | N/A | | |  |  |
| Q.17.66 | | Decline | | Y/N=1/0 | | N/A | | |  |  |
|  | | | | | | | | |  |  |
| Q.18 | | Did you /your child eat a cooked meal that had got cold before your started having loose stools ? | | Answer =[ ] | | | | |  |  |
|  | | *Answer in English* | | *Answer code* | | | *Skip to* | |  |  |
|  | | No, did not do this | | 0 | | | N/A | |  |  |
|  | | Yes, the day the loose stools started | | 1 | | | N/A | |  |  |
|  | | Yes, one day before the loose stools started | | 2 | | | N/A | |  |  |
|  | | Yes, two days before the loose stools started | | 3 | | | N/A | |  |  |
|  | | DK/Do not remember | | 999 | | | N/A | |  |  |
|  | | Decline | | 666 | | | N/A | |  |  |
| Q.19 | | Did you /your child eat a meal where water was added after it was ready cooked before your loose stool started? | | Answer =[ ] | | | | |  |  |
|  | | *Answer in English* | | *Answer code* | | | *Skip to* | |  |  |
|  | | No, did not do this | | 0 | | | N/A | |  |  |
|  | | Yes, the day the loose stools started | | 1 | | | N/A | |  |  |
|  | | Yes, one day before the loose stools started | | 2 | | | N/A | |  |  |
|  | | Yes, two days before the loose stools started | | 3 | | | N/A | |  |  |
|  | | DK/Do not remember | | 999 | | | N/A | |  |  |
|  | | Decline | | 666 | | | N/A | |  |  |
| Q.20 | | What of these following items did you /your child eat between 24 hours before the loose stools started?(Should be allowed multiple answer) | | Answer options with code  *Answer Code:* Y/N=1/0 | | | | |  |  |
|  | | *Name of the foods in English* | | a.Breakfast | b.Lunch | | | c.Dinner | | d.Snacks |
| Q.20.1 | | Rice (rice, pulao, kitchuri, biryani, etc.) | |  |  | | |  | |  |
| Q.20.2 | | Rice soaked in water (panta) | |  |  | | |  | |  |
| Q.20.3 | | Bread (biscuits, shingara, roti, chapatti, naan, etc.) | |  |  | | |  | |  |
| Q.20.4 | | Meat | |  |  | | |  | |  |
| Q.20.5 | | Fish/dried fish | |  |  | | |  | |  |
| Q.20.6 | | Curry | |  |  | | |  | |  |
| Q.20.7 | | Cooked Vegetables (any vegetables, potato or eggplant) | |  |  | | |  | |  |
| Q.20.8 | | Raw Vegetables | |  |  | | |  | |  |
| Q.20.9 | | Eggs | |  |  | | |  | |  |
| Q.20.10 | | Lentils | |  |  | | |  | |  |
| Q.20.11 | | Milk | |  |  | | |  | |  |
| Q.20.12 | | Fruits | |  |  | | |  | |  |
| Q.2013 | | Betel leaf (paan supari) | |  |  | | |  | |  |
| Q.20.14 | | Baby food (other than those not mentioned above) | |  |  | | |  | |  |
| Q.20.15 | | Pickles | |  |  | | |  | |  |
| Q.20.16 | | Did not eat this meal | |  |  | | |  | |  |
| Q.20.99 | | Do not remember | |  |  | | |  | |  |
| Q.20.77 | | Others.................................. | |  |  | | |  | |  |

| Section 5: Information about rectal swab collection | |
| --- | --- |
| Q.21 Have any rectal swab was taken from the person affected with loose stools? | Answer =[ ] |
| *Answer in English* | *Answer code* |
| Yes | 1 |
| No | 0 |
| The person with loose stools was not found in the household within 48 hours | 2 |
| *Declined* | 666 |
| If Declined, specify reason___________ |  |

X. End time of interview: HH : Min

____________________ _______________

Signature of Interviewer Date

____________________ ________________

Checked by Date
