# Supplementary material for: Water usage, hygiene and diarrhea in low-income urban communities—A mixed method prospective longitudinal study
Source: MethodsX. 2019 Nov 19;6:2822–37. doi: 10.1016/j.mex.2019.11.018 (PMC6909126; doi:10.1016/j.mex.2019.11.018)
Supplement: Supplementary file 6 [file mmc6.docx]

# Guideline for in-depth interview

**Perception about seasonality**

1. What different seasons you observe? Please mention names and duration of those seasons.
2. What are the different characteristics of these different seasons?

**Practices related to water use in daily life**

1. List all the personal and household activities that requires water in daily life and explore how each of these practices are affected by water stress.
   1. Water use for personal hygiene/cleaning (e.g. bathing, washing hands, face, legs, washing genital and anus after urination and defecation, and ablution).
   2. Water use for drinking (e.g. plain water, juice, saline, sherbet, tea).
   3. Water use for household hygiene/cleaning (e.g. washing or wiping living/bed room, washing toilet and bathroom, washing water source point, washing cloth, washing kitchen and vegetable and meat cutting place).
   4. Water use for food and food preparation (e.g. washing meat, fish, vegetable, lentil, rice, fruit, betel leaf, washing cooking utensil, glass, plate, adding water in leftover food).
   5. Water use for water container cleaning (drinking water container, water storage container, water carrier).
   6. Reuse of water (for example reuse the vegetable washed water to wash cooking utensil).
   7. Water use for child (e.g. bathing, washing hands, face, legs, washing genital and anus after urination and defecation).
2. Explore the frequency, sources, quantity. Explore the reason while exploring frequency, sources and quantity (For example, usually wipe the bedroom floor once in a day but when child defecate on the floor then wash the floor with water).

**Practice related to water and sanitation during different seasons**

1. Explore changes in frequency, sources and quantity of water use during different season (e.g. summer, rainy season and winter).
2. Explore the changes of above mentioned daily life activities during extreme whether event (e.g. extreme hot, cold and rain).
3. Explore the changes in defecation practices of the informant and children (if any) in different seasons and reasons for changes.
4. Explore the waste disposal (including child feces) practices in different seasons and reasons for changes.

**Perception of water stress and practices/coping strategies during water stress**

1. What problem you face with water (explore if the respondent mentions water stress and link water stress with both quality and/or quantity, ask whether the respondent get enough water for different activities)? What kind of water stress do you have in your household/area? [probe: man-made and natural water stress]
2. Explore if the respondents link water stress with disease occurrence.
3. How many times you face water stress in a year? When? (explore different kind of water stress in different season)
4. Why do you face water stress?
5. What regular activities become interrupted due to water stress? [probe: mandatory, optional and not necessary activities]
6. Explore the decision making about water use during water stress
7. Explore if and how water usage practices changes during water stress to understand the coping strategies (for example, they may compromise with frequency and/or quantity, sources).
8. Explore what kind of loss and disadvantages they experience due to water stress.
9. Explore the coping strategies in terms of community support.
10. Explore the changes in defecation practices of the informant and children (if any) during water stress.
11. Explore the waste disposal (including child feces) practices during water stress.
12. Explore the monthly cost of water source and water supply. Explore if this cost changes over the season throughout the year.

**Perception about diarrhea**

1. What is is diarrhea/loose stool or definition of diarrhea/loose stool? How do you know that you or your family member have diarrhea (explore symptoms)?
2. What do you think about the cause of diarrhea? Why the children gets diarrhea? Why adult gets diarrhea? (probe for example of diarrhea among any of the family member)
3. Who gets diarrhea more frequently- children or adult? Why?
4. Does diarrhea transmit from one person to another? How? [probe: whether the informant understand the fecal-oral transmission routes]
5. Is diarrhea seasonal? What are the seasons (month/s)? Why diarrhea occurs in those certain season?

**Perception about 'cleanliness'**

1. What do you understand about 'cleanliness'?

Personal cleanliness

2. What is personal cleanliness?

3. What are the activities you perform everyday that requires cleaning yourself (list all the activities or times mentioned by the informant)? [probe when your body or hand or face is not clean]

- 1. Among these activities, when do you feel that you must clean yourself/hand/face/leg? Why? [probe: link with religion, people/neighbor will consider it good, husband will like it, aesthetics purposes, child will get sick/good for child health, ablution, menstruation]
  2. When do you feel that cleaning yourself is optional? Why?
  3. When do you feel cleaning yourself is not necessary? Why?
  4. Explore the activities you perform in different seasons that require cleanliness and if there is any change in activities due to seasonal changes [probe: mandatory, optional and not necessary considering each season].

4. When do they wash hand? When hands are visible dirt? After handling child feces, after defecation, before eating food, before feeding baby?

1. From where did you learn about personal cleanliness?
2. What are the agents of cleaning? (water, soap)

Domestic/household cleanliness

1. What is domestic/household cleanliness?
2. What are the activities you perform everyday that requires cleaning of your household floor? probe: when kitchen is not clean, your toilet is not clean, your living room/bedroom is not clean (list all the activities or times mentioned by the informant)
   1. Among these activities, when do you feel that you must clean house, kitchen, toilet, bedroom? Why? [probe: link with religion, people/neighbor will consider it good, husband will like it, aesthetics purposes, child will get sick/good for child health]
   2. When do you feel that cleaning your house/kitchen/toilet/bedroom is optional? Why?
   3. When do you feel cleaning your house/kitchen/toilet/bedroom is not necessary? Why?
   4. Explore the activities you perform in different seasons that require cleanliness and if there is any change in activities due to seasonal changes [probe: mandatory, optional and not necessary considering each season].
3. From where did you learn about domestic/household cleanliness?
4. What are the agents of cleaning? (water, soap, detergent, disinfectant, such as harpic, detol, savlon, phenyl)

Cleanliness of food

1. What food items you or your household members regularly eat?
2. What food items you or your household members eat from outside your home (probe: if they it any raw food/fruits without washing)? Who among your household members eat those more often?
3. Is there any seasonal variation in the food items you or your household members regularly eat?
4. What is clean food and what is unclean food? [probe: cooked food, salad, chilies, lemon, betel leaf]
5. Explore if the informant can perceive the link between food and diarrhea.

Cleanliness of drinking water and other drink

1. What drink items you or your household members regularly or occasionally drink? [probe: drinking water, fruit juice, tea, cold drinks, soda, lemon drink]
2. What drink items you or your household members drink from outside your home? Who among your household members drink those more often?
3. Is there any seasonal variation in the drink items you or your household members regularly drink?
4. What is clean drinking water or drink and what is unclean drinking water or drink?
5. What kind of water do you value the highest drinking purpose? Why (explore whether this choice is linked to hygiene concerns, socio-economic situation, spiritual reason, or other reason)? Explore if there is any difference in source, quantity and frequency of use of drinking water in different season and reason for such change.
6. Explore if the informant can perceive the link between drinking water, other drink and diarrhea.

**Perception about water**

1. What is 'good' water and 'bad' water? Which water is 'good' and which is 'bad'? How do you understand water is 'good' or 'bad'?
2. What is 'clean' water and 'dirty' water? Which water is 'clean' and which is 'dirty'? How do you understand water is 'clean' or 'dirty'?
3. Is there any level of 'goodness/cleanliness' or 'badness/dirtiness' of water?
4. What can you do with 'good/clean' water and what you cannot do with 'good/clean' water?
5. What you cannot do with 'bad/dirty' water? Is there any use of 'bad/dirty' water?
6. Do you clean your water? How do you do it? Do you always have time to clean?
7. Is there any relation between season and the quality of water being 'good/clean' or 'bad/dirty'?
8. Is there any relation between water stress and the quality of water being 'good/clean' or 'bad/dirty'?
